# Enhanced Identifications and Quantification Through Retention Time Down-Sampling in Fast-Cycling Diagonal-PASEF Methods

**DOI:** 10.1016/j.mcpro.2025.101480

**Published:** 2025-12-09

**Authors:** Christopher R. Below, Oliver M. Bernhardt, Stephanie Kaspar-Schönefeld, Sander Willems, Edoardo Coronado, Ino D. Karemaker, Bettina Streckenbach, Monika Pepelnjak, Luca Räss, Sandra Schär, Dennis Trede, Jonathan R. Krieger, Tejas Gandhi, Roland Bruderer, Lukas Reiter

**Affiliations:** 1 Biognosys AG, Schlieren, Switzerland; 2 Bruker Daltonics GmbH & Co. KG, Bremen, Germany; 3 Bruker Belgium NV, Kontich, Belgium; 4 ETH Zurich, Institute of Biochemistry, Zürich, Switzerland; 5 Bruker Ltd, Milton, Canada

**Keywords:** dia-PASEF, diagonal-PASEF, discovery proteomics

## Abstract

Data-independent acquisition (DIA) mass spectrometry is essential for comprehensive quantification of proteomes, enabling deeper insights into cellular processes and disease mechanisms. On the timsTOF platform, diagonal-PASEF acquisition methods have recently been introduced to directly and continuously cover the observed diagonal shape of the peptide precursor ion distributions. Diagonal-PASEF has already shown great promise, and its adaptation as a routine workflow can be further pushed with improved method development as well as enhanced algorithmic solutions. Here, we conducted a systematic and comprehensive optimization of diagonal-PASEF for 17-min gradients on the timsTOF HT in conjunction with Spectronaut. We demonstrate that Spectronaut fully supports all tested diagonal-PASEF methods independent of the number of slices or overlaps and with minimal user intervention required. We derive an optimized analysis strategy where we coupled diagonal-PASEF acquisitions to retention time down-sampling by summation (RTsum) and thereby exploit the fast-cycling nature of diagonal-PASEF methods. Through the combination of RTsum with diagonal-PASEF, we demonstrate that this strategy yields higher signal-to-noise ratios while retaining the peak shape for analytes of interest ultimately improving overall number of peptide and protein identifications of diagonal-PASEF. Importantly, combining RTsum with diagonal-PASEF improved overall identifications and quantitative precision when compared to dia-PASEF with variable quadrupole isolation widths and across different input amounts for cell line injections. We also tested the performance of diagonal-PASEF in controlled quantitative experiments where diagonal-PASEF outperformed dia-PASEF in the overall number of retained candidates below 1% or 5% error-rate, quantitative precision, and identifications on peptide level and protein level. These data indicate that RTsum demonstrates a positive use case of the high sampling rate of diagonal-PASEF and might therefore be a valuable addition to existing analysis pipelines. Collectively, our findings imply that diagonal-PASEF is developing into a competitive alternative to dia-PASEF and that the data analysis options are making fast progress.

## Highlights

• Systematic optimization of diagonal-PASEF on the timsTOF HT with Spectronaut.
• Novel data analysis for diagonal-PASEF with retention time-based down-sampling.
• Diagonal-PASEF is competitive to dia-PASEF and data analysis are developing.
• Diagonal-PASEF is competitive in differential abundance testing.

## Graphical Abstract

**Figure:**
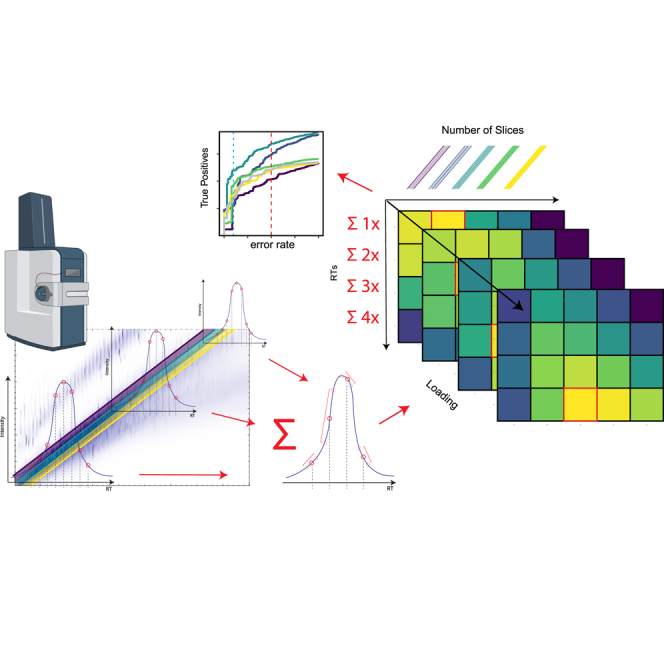

Proteomics studies the complete set of proteins encoded by a genome and as such offers complementary information to the gene dosage governed by nucleic acid expression levels (1). The study of proteins provides mechanistic insights into signaling cascades or drug responses at cellular and even subcellular levels (2).

Historically, proteomics has lagged behind next-generation genome sequencing in terms of scale and depth due to the intricate workflows necessary to accurately capture the multifaceted biochemistry of proteins and peptides. The same biochemical complexity that enables proteins and peptides to drive diverse biology has also necessitated advances in sample preparation, analyte separation and data analysis, all of which presented significant challenges (3). With the improvements in liquid chromatography mass spectrometry-based (LCMS) techniques, in combination with novel bioinformatics technologies, including improved computing power and leveraging artificial intelligence, substantial decreases in data analysis time, as well as increases in proteome coverage, have made high-throughput proteomics not only achievable but also widely adopted (4, 5).

Contemporary LCMS approaches aim to yield high peptide and protein identifications while retaining quantitative accuracy and precision. While data-dependent acquisition (DDA) used to be the gold standard for proteomics, data-independent acquisition (DIA) is increasingly replacing DDA especially for methods with high sample throughput. One of the main advantages of DIA is that it is not dependent on a precursor selection heuristic and thereby results in an unbiased sample survey and higher data completeness by fragmenting each precursor once within each DIA acquisition cycle (6, 7). In addition, precursors as well as corresponding fragment ions provide elution profiles, thus improving the quality of fragment peaks while enabling MS2-level quantitation in addition to that obtained through consecutive MS1 scans. In recent years significant developments in instrumentation, data acquisition and data processing have considerably improved sensitivity and reproducibility of DIA workflows making it ideally suited for high-throughput large sample cohort studies (8, 9, 10). As a result of this, the boundaries of what is possible are continuously shifting in proteomics studies, and this has been reflected in an exponential increase in sample throughput and downscaling of sample preparation to the single cell level (11, 12).

The advent of trapped ion mobility spectrometry (TIMS (13)) technology marked a significant advancement in proteomics, enhancing depth by increasing ion usage through parallel accumulation serial fragmentation (PASEF) (14, 15). TIMS introduces an additional dimension of separation, as well as molecular characterization via collisional cross-sections, thereby providing higher confidence and cleaner MS and MS/MS-spectra (16). Furthermore, TIMS increases selectivity through the spatial and temporal focusing of ions eluting from the ion mobility (IM) device and when applied in conjunction with PASEF, allows for the near complete ion utilization throughout the analytical gradient. By combining DIA with TIMS, sample complexity was shown to be further reduced in a scan mode introduced in 2020 termed dia-PASEF (16). Within dia-PASEF acquisitions, predefined quadrupole isolation windows for ion selection in the mass-to-charge (m/z) and mobility plane are applied in each TIMS elution. A variety of different window schemes have been introduced (17, 18), including the usage of variable window widths (py_diAid (19)), very narrow windows (thin-dia-PASEF (20)) and application of a method that continuously scans precursors with multiple horizontal quadrupole isolation windows (slice-PASEF (21)). In addition, such methods have also been used in conjunction with scan summation, which has the promise to improve the signal-to-noise ratio for single-cell-based analyses of fast-scanning timsTOF data (12, 21).

To further increase the analytical performance of DIA-based approaches on timsTOF mass spectrometers, researchers have recently proposed diagonal-PASEF acquisition methods termed either synchro- or midia-PASEF, which were designed to acquire the ion cloud more efficiently (22, 23). These methods operate by seamlessly and continuously following the observed diagonal shape of the precursor ion distribution in m/z and ion mobility dimension. The quadrupole isolation window is moved synchronously with the IM elution, which leads to better utilization of the trapped ion cloud and allows correlation of the fragment and precursor signal more tightly. The synchronized movement can slice the ion cloud into several, method-dependent, TIMS ramps, which are often termed diagonal-PASEF slices. Compared to conventional dia-PASEF approaches, the cycle time of diagonal-PASEF methods does not increase with an increased overall mass range, as each scan covers the desired m/z range. This is particularly true for non-overlapping schemes, such as synchro-PASEF, where only a few slices are required to cover the range of interest, thereby resulting in methods with very short cycle times and improved peak coverage as a result of more frequent sampling.

Initial studies have shown that synchro-PASEF can achieve comparable proteome depth compared to classic dia-PASEF modes with superior quantitative performance as a result of the higher sampling rates, which demonstrates the potential of diagonal-PASEF acquisition modes (18, 22). Software tools have already integrated initial data analysis algorithms for diagonal-PASEF (Spectronaut, DIA-NN, and alphaDIA), and initial results show that such acquisition methods promise to cover the observed ion-cloud more efficiently. Their impact and more widespread adaptation can likely be improved by systematic method evaluation and enhanced data processing over the current state. As such several parameters could be further optimized to tailor diagonal-PASEF methods for specific applications and thus improve their real-world applicability. Effective implementations could focus on maximizing sensitivity and minimizing cycle time for optimal peak coverage. Scan summation strategies seem particularly interesting for such attempts and have been described for dia-PASEF and slice-PASEF applications (12, 21). Therefore, multiple lines of evidence indicated that scan summation could hold the promise to improve the signal-to-noise ratio of acquisitions, particularly for single cell-based analyses of fast-scanning timsTOF data (12, 21). Unfortunately, to date, such approaches have not been applied to fast-scanning diagonal-PASEF methodologies. We therefore set out to develop a data analysis algorithm with optimized ion current extraction and scan summation for Spectronaut which would allow us to systematically apply scan summation to diagonal-PASEF data (6). We optimized the diagonal-PASEF acquisitions on a timsTOF HT mass spectrometer for different applications and provided evidence for its benefit in modern proteomics applications.

## Experimental Procedures

### Protein Material from Cell Pellets

HeLa cell pellets were purchased from Cell Line Services. *Escherichia coli* and *Saccharomyces cerevisiae* digests were kindly provided by Dr Audrey van Drogen*. Caenorhabditis elegans* Bristol strain worm digests were kindly provided by Prof. Monica Gotta (University of Geneva). Frozen HeLa cell pellets were purchased from Dundee cell products.

### Sample Preparation

HeLa cell pellets (Cell Line Services) were diluted in 100 μl of Biognosys lysis buffer and subsequently lysed and homogenized using a LE220Rsc ultrasonicator (Covaris). Following sonication, samples were denatured at 95 °C for 5 min. All subsequent liquid handling and sample processing were automated using a Hamilton Microlab STAR system, equipped with a HMotion robotic arm, Hamilton heater shakers, and integrated third-party devices, including an Epoch microplate reader (Agilent), Ultraseal Pro plate sealer (Porvair Sciences), Xpeel plate peeler (Azenta), and Ultravap Mistral blow-down evaporator (Porvair Sciences). Protein concentrations were determined using the BCA assay (Thermo Fisher Scientific) according to the manufacturer’s instructions. For further processing, 75 μg of protein was used in a protein aggregation capture (PAC) protocol, as described previously (3, 24, 25). Peptide desalting for mass spectrometry was performed using an Oasis HLB μElution Plate (30 μm, Waters) following the manufacturer’s protocol. Desalted peptides were dried using a blow-down evaporator and reconstituted in LC solvent A (1% acetonitrile in water with 0.1% formic acid) containing Biognosys’ iRT-peptide mix for retention time calibration. Peptide concentrations in mass spectrometry-ready samples were measured using the mBCA assay (Thermo Fisher Scientific). For loading ramp experiments, the peptide digest was diluted in LC Solvent A to a final concentration permitting the infusion of 1 μl sample material per acquisition.

Mixed proteome samples for quantitative controlled experiment (CQE) were prepared as previously described (7). In brief, the *C. elegans* worms were first lysed using a bead mill (Eppendorf) upon suspension in 8 M urea and 0.1 M ammonium bicarbonates and thereafter subjected to preparation using the filter aided sample preparation protocol following by desalting using the Oasis HLB μElution Plate (30 μm, Waters). *E. Coli,* HeLa, and *S. cerevisiae* samples were re-suspended in 8 M urea lysis buffer, vortexed, and the protein concentration was determined using the BCA assay (ThermoFisher) according to the manufacturer’s instructions. Samples were then reduced (10 mM Tris(2-carboxyethyl)phosphine) and alkylated (40 mM CAA Chloroacetamide) at 800 rpm, 37 °C for 60 min followed by a digestion with trypsin (Promega) in a 1:50 ratio overnight at 800 rpm, 37 °C. Prior to digestion the 8 M urea buffer was diluted using 0.1 M ammonium bicarbonate to dilute the urea to 2 M. Upon digestion, samples were desalted using the HLB 96 well-plates (Waters) according to the manufacturer’s instructions and peptide concentration was determined using the mBCA kit (ThermoFisher Scientific). Proteomes were mixed in the following ratios*: H. Sapiens:E.coli:S.cervisiae:C.elegans* at 1:10:2.1:0.77. For loading ramp experiments, the peptide digest was diluted in LC Solvent A to a final concentration permitting the infusion of 1 μl sample material per acquisition.

### Liquid Chromatography Coupled Mass Spectrometry

Unless otherwise indicated, 800 ng of peptides were injected to an IonOpticks Aurora series Ultimate CSI 75 μm × 250 mm C18 reversed phase column (AUR3-25075C18-CSI) on a Thermo Scientific EASY-nLC1200 nano-liquid chromatography system connected to a Bruker Daltonics timsTOF HT mass spectrometer equipped with a Captive Spray II ion source. LC solvents were A: water with 0.1% FA; B: 80% acetonitrile, 0.1% FA in water. The nonlinear LC gradient was 1 to 45% solvent B in 18 min followed by a column washing step in 90% B for 2.5 min, and a final equilibration step of 1% B for 3 min at 60 °C with a flow rate set to a ramp between 600 and 400 nl/min (min 0: 600 nl/min, min 18: 400 nl/min, washing at 600 nl/min) as follows: Time[min]:%B[%]:Analytical Flow[μL/min]: 0:1.0:0.6, 1:6.0:0.60, 2:8.0:0.6, 3:10.0:0.6, 4.16:11:0.58, 5.33:13:0.57, 6.5:15:0.56, 7.7:16.0:0.53, 8.83:18:0.52, 10:20.0:0.511, 11.33:21:0.49, and 18:45:0.4. For HeLa or mixed proteome loading ramp experiments, the indicated loading amount was injected onto the analytical separation column. All acquisitions were conducted in positive ionization mode at a source capillary voltage of 1500 V. During the operation of the instrument, every acquisition had in-batch calibration enabled, whereby the Tuning MIX ES-TOF CCS reference list was used as calibration template. Automatic calibration was conducted on calibrants with m/z of 622.029, 922.0098, 1221.9006 (Chip Cube High Mass Reference kit, Agilent Technologies).

### Method Design of Dia-PASEF and Diagonal-PASEF Methods

All dia-PASEF and diagonal-PASEF methods consisted of one full range MS1 scan from 100 to 1700 m/z with an applied ion mobility range from 0.7 to 1.45 1/k_0_ (inverse reduced ion mobility) with ramp and accumulation times set to 70 ms (100% duty cycle). Default processing and TIMS settings were applied for all methods; the mobility detection threshold was set to 5000, and no denoising was applied to any acquisition. For all acquisitions, the applied collision energy was set to a custom ramp between 0.8 and 1.45 1/k_0_ as follows: [1/k_0_:eV]: 0.8:30, 0.95:30, 1.05:35, 1.15:45, 1.25:55, 1.35:65, and 1.45:55. The “Base” type was used for any stepping in the custom gradient.

The dia-PASEF method was generated using an in-house software (Biognosys AG) and consisted of 12 dia-PASEF ramps resolved over 50 boxes with variable width and a mass overlap set to 0.5 Th between adjacent boxes. The effective m/z coverage of dia-PASEF boxes was 175 to 1450, and the method resulted in a cycle time of 0.99 s.

The dia-PASEF ‘py-diAID’ method was generated using the “py_diAID” tool using the “Optimal dia-PASEF” configuration in default mode (18, 19). The desired 1/k_0_ range was set to 0.7 to 1.45 at 12 PASEF ramps, with mass overlap set to 1 Th between adjacent boxes, and 100 iterative optimization steps were conducted with the number of starting points set to 20. The optimal method consisted of 24 boxes resolved over 12 PASEF ramps and allowed for the highest theoretical precursor coverage (88.19%).

The diagonal-PASEF methods were generated using the timsControl diagonal-PASEF Window Editor (timsControl V6.06, Bruker Daltonics GmbH & Co. KG). For all HeLa and mixed proteome loading ramp experiments the width of the diagonal-PASEF beam was set to 200 m/z and resolved by 1, 2, 4, eight or 12 slices, leading to an effective isolation width per slice of 200, 100, 50, 25, or 16.67 m/z. The resulting m/z start and end ranges were constant for all diagonal-PASEF methods and resulted in an m/z start of the slices between 375 and 1250 m/z and an m/z end of 567 m/z and 1447 m/z. All diagonal-PASEF methods had an effective overlap of the slices of 1 Th as previously described elsewhere (23). For the implementation of the overlap between slices, a midia-PASEF license was enabled and utilized in the timsControl software. The cycle time of the methods varied depending on the number of diagonal-PASEF slices. For the ramping of the diagonal-PASEF slices, the diagonal-PASEF slices varied between 1 and 20 slices, and an overlap of the slices was disabled for this experiment.

### Diagonal-PASEF Calibration

All diagonal-PASEF methods were calibrated as per the recommendations by Bruker. In brief, upon successful calibration of the m/z and the mobility of the timsTOF HT, each diagonal-PASEF method was loaded, and the ESI-L Low Concentration Tuning Mix (Agilent Technologies) was infused at 3 μl/min through the ESI Apollo source. The diagonal-PASEF quadrupole calibration routine was conducted, and the methods were calibrated on the Background ions while the ions from the calibration mix were excluded through the Tuning Mix ES (ESI) exclusion list. Diagonal-PASEF methods were calibrated on a bi-weekly basis to ensure sufficient isolation of the quadrupole.

### Mass Spectrometric (MS) Data Analysis

All dia-PASEF and diagonal-PASEF data were analyzed using Spectronaut version 20.2 (Biognosys AG, Schlieren, Switzerland). The directDIA pipeline was used for all acquisitions and conditions were subjected to individual searches whereby all replicates belonging to one search were grouped. For all analyses, the modified default settings were used as described by the manufacturer (Biognosys AG). In brief, Carbamidomethylation (C) was used as fixed modification and Acetyl (Protein-N-term) and Oxidation (M) were used as variable modifications. The PSM, Peptide, and Protein group false discovery rates (FDR) were set to 1% during the Pulsar search. No imputation was applied during the quantification of the analytes, and minor (peptide) grouping was conducted on Stripped Sequence level. The following modifications were applied: For dia-PASEF acquisitions, the dia-PASEF pre-processing mode “Automatic” was used, IM sampling reduction was set to seven and RT sampling reduction was set to 1. For diagonal-PASEF acquisitions, the dia-PASEF pre-processing mode “Legacy (Spectronaut 18)” was used, IM sampling reduction was set to three and RT sampling reduction was set to the indicated value in the main text (1–4). To perform RT down sampling, multiple consecutive scans of the same scan definition can be combined into a merged scan. A set of scans that need to be merged are combined into a list of m/z intensity pairs and ordered by m/z in ascending order. Starting from the smallest peak, peaks within a tolerance of 15 ppm are combined into a new combined ion with an intensity weighted averaged m/z, averaged retention time, and summed intensity. For the generation of representative extracted ion mobilogram (XIM) plots a 100 ng HeLa injection conducted with dia-PASEF or diagonal-PASEF was utilized, and the data searched by setting the IM summation to one and dia-PASEF pre-processing set to “Fast (Spectronaut 20)” for dia-PASEF and diagonal-PASEF. For the systematic testing of the optimal search and analysis settings for diagonal-PASEF data, the values were used as indicated in the main text. FASTA files were generated using the UniProt sequence database, the following FASTA files were used: For HeLa acquisitions: *Homo sapiens* Uniprot/Swiss-Prot database from 2024-07-01 containing 20,435 entries, for mixed proteome acquisitions: *H. sapiens* Uniprot/Swiss-Prot database from 2024-01-01 containing 20,418 entries, *S. cerevisiae* (strain ATCC 204508/S288c) Uniprot/Swiss-Prot database from 2024-01-01 containing 6727 entries, *E. coli* (strain K12) Uniprot/Swiss-Prot database from 2024-01-01 containing 4530 entries, *C. elegans* Uniprot/TrEMBL databased from 2024-01-01 containing 27,453 entries. For all FASTA files in mixed proteome experiments, only the peptide sequences that are unique to each species were retained. *Arabidopsis thaliana* Uniprot/Swiss-Prot database from 2025-01-01 containing 16,394 entries (Peptides mapping to human proteome were removed).

### Experimental Design and Statistical Rationale

All data analyses were conducted in the R programming language (V. 4.5.1) using the tidyverse (V. 2.0.0) package environment (26) for all data handling and modification in RStudio (V. 2025.05.1 + 513). Prior to data handling, the quantitative data were extracted on peptide precursor level from Spectronaut using a custom report schema. Correlation analyses were conducted using curing the cor.test (27) function in R and “Spearman” or “Pearson”-type correlation were applied on log10-transformed MS2 quantities of analytes of interest depending on the normality of the datasets as indicated. Normality of the data was assessed on non-transformed data through the Shapiro.test function in R (28). For the correlation analysis of different acquisition methods, the chart.correlation function was utilized from the PerformanceAnalytics package (V. 2.0.8) in R. For the computation of any data points per peak (DPPP) value, we utilized the “EG.DatapointsPerPeak” column from Spectronaut. For the computation of DPPP, Spectronaut counts the number of raw datapoints that fall within the peak integration boundaries. Peak width is defined as FWHM ∗ 1.7. This means that any tailing points are not counted as data points. For the computation of the signal-to-noise ratio we divided the quantity (“EG.Quantity”, Spectronaut) value of each precursor by the noise (“EG.Noise”, Spectronaut) value. The number of MS1 or MS2 spectra was obtained from the columns “R.MS1Spectra” or “R.MS2Spectra” from Spectronaut. Upset plots were generated using the function upset from the UpSetR (V. 1.4.0) package in R on binarized data on the desired analyte level. Only the 20 interactions with the highest frequency were included. One-sided ANOVA was conducted using the aov function in R, and pairwise comparisons were conducted using Tukey’s ‘Honest Significance Difference’ method through the TukeyHSD function in R. Quantitative peptide precursor-level data were exported from Spectronaut including for the CQE experiments. Subsequently, using R, the data were collapsed to the desired quantitative unit (peptide stripped sequence or protein groups). The data were normalized such that the median of the stable human background proteome was equal for all acquisitions. Next, quantitative units that have zero variance or were <100 intensity were removed to avoid the target identifications to be driven by noise. The quantitative data were log-transformed. Statistical analysis was performed on normalized MS2 quantitative level using a two-sided *t* test assuming equal variance. FDR was calculated using the Storey approach (29). The fold change was calculated based on log2(condition 2)/log2(condition 1). Next, the ground truth was annotated, human as “background” and the other organisms as “spike-in” with a theoretical fold change column. Subsequently, the data were sorted by ascending *p*-value and then the true positives and true negatives were counted. All the regulated candidate proteins are sorted by *p*-value and categorized as true and false positives based on the ground truth pipetting. Finally, the number of true candidates can be calculated based on the error rate of finding the control species. We tested the empirical FDR using two different entrapment protein databases: (1) Using a cleaned up *A. thaliana* database as a decoy species, removing the peptides that map to human database, and (2) Using a synthetic entrapment database as described in Wen *et al*. Nature methods (2025) (30) and further mutating the C-terminal amino acids of every tryptic peptide (K- > R and R- > K). As false positives, we counted the proteins that contained at least one peptide uniquely mapping to entrapment FASTA based on PEP.AllOccuringOrganisms column. We calculated the lower bound, the conservative upper bound, and the tight upper bound (paired) as described in Wen *et al*. Nature method (2025) (30). For the evaluation of RT summation on previously published data, we obtained the raw acquisitions and search data from Skowronek *et al* Nature Protocols 2025 via MassIVE (Data identifier: ftp://massive-ftp.ucsd.edu/v07/MSV000094389/) (18). For Spectronaut results, we searched the data in Spectronaut 20.2 using the indicated settings and RT summation values in directDIA mode. The following number of replicates and total comparisons were performed in each experiment: Experiment 1 (optimization of Spectronaut parameters “DIA pre-processing” and “Ion Mobility down-sampling”, one diagonal-PASEF acquisition was utilized (n = 1); Experiment 2 (assessment of optimal diagonal-PASEF slices), three distinct acquisitions were performed per tested number of slices (n = 3); Experiment 3 (assessment of RTsum for diagonal-PASEF data): four distinct acquisitions were performed for the 2-slice method at 10 and 100 ng of loading (n = 4), for ANOVA testing of impact of RTsum on signal-to-noise ratio nine comparisons were conducted in total (no sum vs 2, three or 4×; 2× vs 3×, 4×; 3× vs 4×); Experiment 4 (loading ramp of diagonal-PASEF and dia-PASEF on HeLa): four distinct acquisitions were conducted for each loading and method (n = 4); Experiment 5 (loading ramp of controlled quantitative experiment): three distinct acquisitions were conducted for each loading, method and sample (A or B) (n = 3).

## Results

### Spectronaut Supports Diagonal-PASEF Acquisitions

**Fig. 1:**
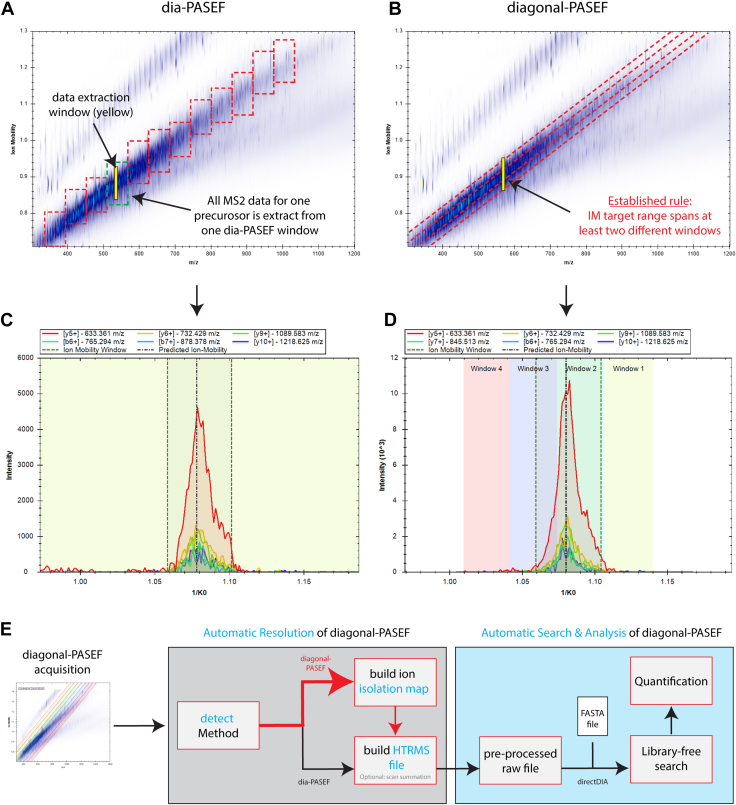
**Spectronaut allows for the seamless integration of diagonal-PASEF workflows into existing analysis pipelines.***A-B*, schematic overview of dia-PASEF (*A*) or diagonal-PASEF (*B*) acquisition methods across an exemplary ion distribution. *Yellow* areas indicate a schematic data extraction window designated for targeted MS2 data extraction. *C-D*, extracted ion mobilogram (XIM) of a representative precursor “_DLYEDELVPLFEK_.2” on MS2-level from a dia PASEF (*C*) or diagonal-PASEF (*D*) acquisition. Individual fragments are depicted as differentially colored lines. Data extraction range is depicted as *dashed vertical lines*. Isolation windows governed by the acquisition method are shown with a coloured background. *E*, simplified schematic visualization of a directDIA search and analysis workflow implemented in Spectronaut 19 or later for diagonal-PASEF data.

To systematically test diagonal-PASEF LCMS methods, we first had to extend the data analysis of Spectronaut to support this novel type of data. The main aspect of making Spectronaut support diagonal-PASEF was to change the data-extraction region that is used to extract MS2-features for any given MS1 event. In Spectronaut version 18, the predecessor of Spectronaut version 19 and 20, the MS2-features for any given precursor are only extracted from one dia-PASEF box that is defined as rectangle in 1/k_0_ – m/z space (Fig. 1*A*). As diagonal-PASEF conducts continuous quadrupole movements, such rectangles are not applicable for these types of methods. We therefore adapted the data extraction algorithm to account for individual isolation windows per microscan within a diagonal-PASEF frame (Fig. 1*B*). This was necessary because every microscan has a different parent isolation. To account for this complexity, an isolation map is created by grouping microscans by their respective slices with each MS2 ion annotated by the corresponding MS1 isolation window allowing for an efficient querying of the data. To validate that the MS2-extraction worked sufficiently with the new data extraction rule in Spectronaut version 19 and 20, we investigated extracted ion mobilograms (XIMs) from precursors that were identified in a HeLa acquisition using either a diagonal-PASEF MS-method with 4-slices or a dia-PASEF MS-method consisting of 12 PASEF-ramps. We investigated the protein HNRPR (Uniprot accession O43390) for which 48 unique precursors were identified in the diagonal-PASEF run and 51 were identified in the dia-PASEF acquisition (Fig. 1, *C* and *D*, Supplemental Fig. S1*A*). Manual inspection of representative XIMs generated from MS/MS scans highlighted the absence of any artifacts or data-gaps, especially between adjacent slices for the investigated diagonal-PASEF acquisition (Fig. 1*D*). In addition, the XIM of the selected precursor did not show any alterations between the dia-PASEF or diagonal-PASEF acquisition, albeit a higher intensity signal was detected, which confirmed previous findings from other studies (22). We validated these findings in two additional precursors “_NLATTVTEEILEK_.2” which also mapped to HNRPR (Supplemental Fig. S1*B*) and “_DLLDLLVEAK_.2” which mapped to DDX3X (Uniprot accession O00571, Supplemental Fig. S1*C*). These data indicated that an efficient data usage can be conducted using the upgraded data-extraction routines. To more easily process diagonal-PASEF data, we next built an automatic method detection algorithm into all Spectronaut versions from version 19 (Fig. 1*E*). Using this algorithm, Spectronaut will automatically determine any PASEF-based data-independent acquisition type and apply the correct downstream processing, thus making seamless analysis of diagonal-PASEF acquisitions possible without any manual intervention of the user.

To further optimize the processing of diagonal-PASEF data, we next investigated the suitability of the applied Pulsar search & DIA-analysis settings which are applied during the directDIA pipeline (Supplemental Fig. S1, *D–F*). For this we investigated the effect of the IM down-sampling by summation (IMsum) and DIA pre-processing settings on the precursor identifications for a representative diagonal-PASEF acquisition. We found that an IM summation value of three together with the DIA pre-processing set to “Legacy (Spectronaut 18)” mode improved the precursor, peptide and protein group identifications compared to the “BGS Factory Settings” which we deemed optimal for dia-PASEF acquisitions. During the “Legacy” pre-processing routine, Spectronaut will consider all micro-scans on the MS2 level that fall within the parent MS1-feature over the full IM width whereas in the “Spectronaut19 pre-processing” routine, only micro-scans that correspond to the IM-apex are considered. The usage of the MS2 apex micro scan only would significantly lower the number of ions used for the construction of the pseudo-DDA spectra during the spectrum centric search for diagonal-PASEF, which is why we deviated from using this strategy. We validated that the FDR in Spectronaut is well-controlled for diagonal-PASEF acquisition using a published entrapment methodology (Supplemental Fig. S1*G*) (30).

Taken together, with the improved data-extraction of the Spectronaut algorithm, we were able to upgrade the software to fully support various diagonal-PASEF acquisitions, including synchro- and midia-PASEF while optimizing identification performance.

### Systematic Optimization of Diagonal-PASEF Acquisitions for Short Gradients

**Fig. 2:**
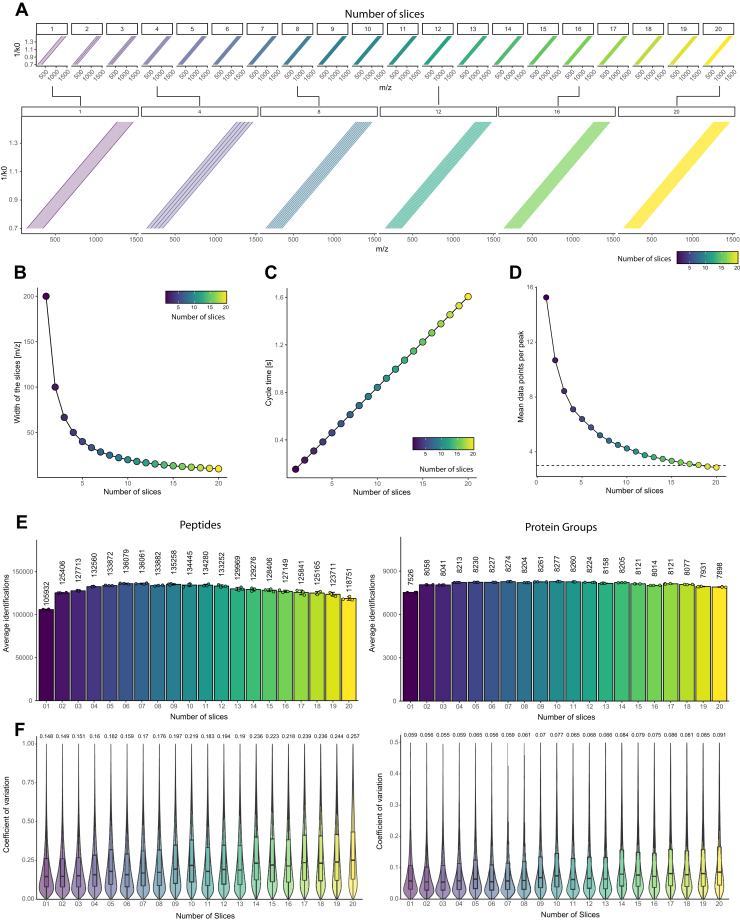
**Diagonal-PASEF measurements and data-analysis are stable across a wide range of tested methods.***A*, depiction of tested diagonal-PASEF methods for method screening approach. *Top*: Methods ordered by ascending number of slices ranging from 1 to 20. All tested methods had an overall width of 200 m/z. *Bottom*: Magnification of selected methods. *B-C*, width of slices in m/z (*B*) and resulting cycle time in s (*C*) for all tested methods from *A*. Each *dot* represents a single method. *D*, data points per chromatographic peak (DPPP) of all tested methods from A in 17-min gradients. Mean and standard deviation of mean shown (n = 3). Horizontal *dashed line* represents 3 DPPP. *E*, HeLa peptide (*left*) or protein group (*right*) identifications of all diagonal-PASEF methods from A in 17 min gradients when 800 ng are injected. Mean identifications are shown. *Error bars* indicate standard deviation of mean (n = 3). *F*, coefficient of variation (CV) of all peptide (*left*) or protein group (*right*) identifications from (*E*). *Boxplots* indicate the inter-quartile range (IQR) from the lower quartile to the upper quartile. *Central line* indicates the median value of the population and whiskers indicate the 1.5 × IQR. Median CV is indicated above the plot. Outliers are not shown to aid the visual interpretation of the data.

Next, we investigated the performance of diagonal-PASEF in proteomics LCMS acquisitions in conjunction with our optimized Spectronaut analysis strategy. One of the key metrics of any MS-acquisition on the timsTOF systems is the cycle time of an acquisition method. The cycle time is determined by the number of PASEF ramps, which are conducted at a defined ion mobility elution (or ramping) time. Here, we used the timsTOF HT mass spectrometer and operated the system in a way that we kept the ramp and accumulation times at 100 ms. Given the selected chromatography, which generated peaks with a full width at half the maximum of about 6 to 12 s, depending on the gradient length, we decided to generate diagonal-PASEF MS methods ranging from a single diagonal-PASEF slice to twenty diagonal-PASEF slices (Fig. 2*A*). To ensure comparability of the methods and allow for an optimal sampling of the ion-cloud, we kept the width of the methods constant at 200 m/z for all tested methods. Due to this fixed overall method width, the resulting diagonal-PASEF method varied in the width of the individual slices and ranged from 200 m/z for the single-slice method to 10 m/z for the 20-slice method (Fig. 2*B*).

The performance of diagonal-PASEF was examined by applying the generated MS methods in analytical triplicate injections of 800 ng of a HeLa sample in 17-min gradients (∼37 samples per day) (Fig. 2, *C*–*F*). The fastest cycling method, consisting of a single slice, had a cycle time of 0.15 s (Fig. 2*C*) and allowed for sampling the chromatographic peak on average 15.6 times in 17-min gradients (Fig. 2*D*). In comparison, the 20-slice diagonal-PASEF method had a cycle time of 1.61 s and achieved 2.8 DPPP on average. We next investigated the peptide and protein group identifications that we obtained from the diagonal-PASEF acquisitions in directDIA searches in Spectronaut version 20 (Fig. 2, *E* and *F*). Spectronaut processed all tested diagonal-PASEF acquisitions seamlessly, indicating that the software is suitable to analyze diagonal-PASEF methods with vastly different numbers of slices and sampling rates. We observed that the obtained average number of peptides varied strongly across the tested methods and gradient lengths (Fig. 2*E*). The method yielding the highest peptide identifications (136,079) consisted of six slices, whereas most protein group identifications (8277) were achieved when using a 10-slice method. The difference between the best- (6-slice) and worst-performing (1-slice) methods was 28.5% on peptide level, whereas on the protein group level, this difference was 9.9% (10-slice vs 1-slice). In general, the number of identified protein groups was highly stable with acquisition methods between 4-slices and 12-slices showing a standard deviation of only 35.8 protein groups, while on the peptide level, the identifications varied strongly between the tested methods. This data indicated that diagonal-PASEF methods with different numbers of slices are more variable on the peptide level and to a lesser extent on the protein level, which is likely due to the coverage of proteins by multiple peptides.

Next, we investigated the quantitative precision of the diagonal-PASEF methods. For this, we evaluated the median coefficient of variation (CV) of all methods that were acquired (Fig. 2*F*, Supplemental Fig. S2, *A* and *B*). We observed that the median CV on the peptide level generally increased with an increase in the number of diagonal-PASEF slices from 14.8% at 1-slice to 25.7% with methods consisting of 20 diagonal-PASEF slices. On the protein group level, we observed similar trends and found the median CV to increase from 5.9% when using the 1-slice method to 9.1% when using the 20-slice method. When investigating the median CV over the DPPP we noticed that methods with >4 DPPP generally had a lower median CV compared to methods with <4 DPPP and that the median CV markedly increased with methods having more than 4 DPPP (Supplemental Fig. S2, *C* and *E*). Given that the maximum peptide and protein group identifications with diagonal-PASEF methods were achieved when using 4 to 12 slices methods and that the lowest medium CV was achieved with methods having the least slices, we next investigated the identifications below 10% or 20% CV to identify the methods with the best quantitative precision (Supplemental Fig. S2, *D* and *F*). This analysis revealed that a 6-slice diagonal-PASEF method achieved the highest quantitative precision with 80,327 (58.2%) and 43,255 (31.3%) of all peptides quantified with <20% and <10% CV, respectively. On the protein level, while most proteins were quantified with a 10-slice method, the 6-slice and 2-slice methods yielded the most identifications <20% (7359) and <10% CV (5915) respectively. These data indicate that an increase in the number of DPPP did not yield more peptide identifications below 20% or 10% CV indicating that for diagonal-PASEF methods a balance between the number of diagonal-PASEF slices and the cycle time needs to be reached to achieve best performance.

### Retention-Time Summation Improves the Signal-To-Noise Ratio of Diagonal-PASEF Acquisition

**Fig. 3:**
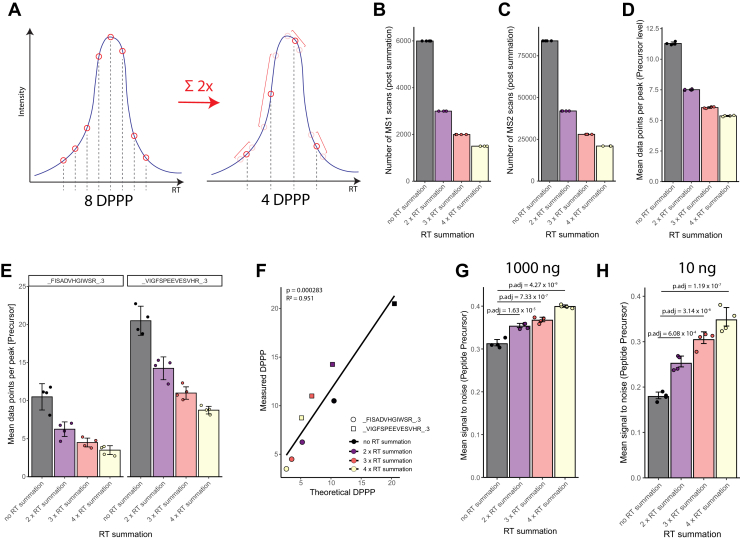
**Retention time down-sampling summarizes adjacent MS or MS/MS scans and thereby enhances the signal-to-noise ratio of diagonal-PASEF data.***A*, schematic illustrating the concept of retention time summation. *B–D*, characteristics of a 2-slice diagonal-PASEF method subjected to 1 to 4 x retention time summation. Data from 1000 ng HeLa acquisitions in 17-min gradients. *B*, number of MS1 scans per injection post retention time summation. *C*, number of MS2 scans per injection post retention time summation. *D*, mean data points per chromatographic peak per injection post retention time summation shown. *Error bars* indicate standard deviation. *E*, mean DPPP for selected precursors for a 2-slice diagonal-PASEF method with indicated retention time summation. *Error bars* indicate standard deviation of mean. Individual datapoints indicate replicate acquisitions. *F*, pearson-based correlation of the empirically measured DPPP and the theoretically expected DPPP for the two selected precursors from *E* across tested retention time summation values. *Diagonal line* indicates the linear regression of the measured and theoretically determined DPPP. Correlation coefficient and correlation *p*-value are shown. *G-H*, mean signal-to-noise ratio on precursor level per injection shown post retention time summation for 1000 ng (*G*) or 10 ng (*H*) input during the acquisition. *Error bars* indicate standard error of mean. Adjusted *p*-values from two-sided Tukey HSD test upon computation of one-sided ANOVA *p*-value (1000 ng: *p* = 8.06 × 10–9; 10 ng: *p* = 1.54 × 10–7). Selected pairwise comparisons are indicated.

The data obtained so far in this study indicated that diagonal-PASEF methods can result in a high peak coverage with elevated number of DPPP due to the fast-sampling rate of the methods that appears to yield a high quantitative precision of such methods. We therefore next sought to exploit this possible over-sampling and subjected the 2-slice diagonal-PASEF method to a retention time down-sampling strategy (Fig. 3*A*, Supplemental Fig. S3). We chose the 2-slice method as it achieved the highest number of protein groups quantified <10% CV at a high number of DPPP and thus was a good model method to evaluate whether down-sampling of the scan could further boost the performance of such fast-cycling methods. Similar strategies have previously been conceptualized for dia-PASEF and more recently slice-PASEF applications but have never been realized for diagonal-PASEF-type methods (12, 21). Here, we applied the concept of retention-time down-sampling by summation (RTsum) subsequent to the ion mobility summation (IMsum) which is already conducted within the Spectronaut analysis pipeline. In the IMsum approach multiple dia-PASEF scans are down-sampled in the 1/k_0_ dimension to improve the signal-to-noise ratio of the derived XIM. In Spectronaut, RTsum can be applied during the pre-processing of the search & analysis pipeline of any dia-PASEF or diagonal-PASEF data (materials & methods). Commonly, the RTsum value can be set as any strictly positive integer and determines the number of adjacent MS1 and MS2 scan events that are summarized during the processing of the run. During these summarizations the effective RT of the scans will be averaged, as such if a RTsum value of two is chosen for an acquisition that yields 8 DPPP then the resulting acquisition will be rendered to 4 DPPP whereby every second adjacent MS1 or MS2 scan is summarized into a novel summarized scan (Fig. 3*A*). We next validated the effects of RTsum on a 2-slice diagonal-PASEF acquisitions of 1000 ng of a HeLa sample acquired in 17-min gradients (n = 4) and tested summation values ranging from 1 (no-summation) to 4 (4× summation, Fig. 3, *B*–*G*). As expected, we found the number of MS1 (Fig. 3*B*) or MS2 (Fig. 3*C*) scans post analysis in Spectronaut to decrease in direct proportion to the defined RTsum value. We also identified the mean DPPP for all precursors identified in a single acquisition to decrease in direct proportion with the utilized RTsum value although a buffering effect was observed for an RTsum value of three and 4 (Fig. 3*D*). We reasoned that this buffering might arise from populational effects as not all precursors will have exactly the same peak width throughout the analytical gradient (Supplemental Fig. S3*A*, (31, 32)). To more accurately investigate the effect of RTsum on the peak shape, we assessed the extracted ion chromatogram (XIC) and DPPP of the peptide-precursors FISADVHGIWSR (3+) and VIGFSPEEVESVHR (3+) (Fig. 3*E*, Supplemental Fig. S3 *C* and *D*). Without RTsum, the 2-slice diagonal-PASEF method sampled the chromatographic peak of FISADVHGIWSR (3+): 10.5 and VIGFSPEEVESVHR (3+): 20.5 times on average across all four replicates. When applying an RTsum of 2, three or four the DPPP for FISADVHGIWSR (3+) were reduced to 6.25 (40.5% reduction), 4.5 (56.7% reduction) and 3.5 (66.7% reduction) respectively. For VIGFSPEEVESVHR (3+) applying an RTsum of 2, three or four reduced the DPPP to 14.2 (30.7%), 11 (46.3%) and 8.75 (57.3%) respectively. There was a significant correlation (*p* = 0.000283; R^2^ = 0.95) between the measured and the theoretically expected DPPP after RT summation, indicating that RTsum does not introduce DPPP artifacts (Fig. 3*F*). To validate that RTsum does not influence the peak-shape or chromatogram extraction, we next investigated the XIC of the selected precursors and could indeed not observe any alterations of the XIC shape on the MS1 level for the mono-isotopic, M + 1 or M + 2 envelope (Supplemental Fig. S3*C*). In summary, these data provide evidence that the RT summation strategy provides the expected results and does not yield any sampling or XIC biases on the resulting data.

To investigate putative benefits of RTsum on the diagonal-PASEF data, we investigated the average signal-to-noise ratio for all peptide precursors identified with a 2-slice diagonal-PASEF method (Fig. 3*G*). Strikingly, we identified that using an RTsum of 2 (adj. p-val = 1.63 × 10^−5^), 3 (adj. p-val = 7.33 × 10^−7^) or 4 (adj. p-val = 4.27 × 10^−9^) provided a significantly higher signal-to-noise ratio compared to when not applying any RTsum (ANOVA *p*-value = 8.06 × 10^−9^) which we quantified by dividing MS2 quantity of each precursor by its respective noise level (materials & methods). We confirmed these findings through injecting 10 ng of a HeLa material using the 2-slice diagonal-PASEF method and subjected the resulting data to no RTsum or RTsum of 2, 3 or 4 (Fig. 3*H*, Supplemental Fig. S3*B*). We observed that RTsum significantly improved the signal-to-noise ratio for all detected peptide precursors compared to not applying any retention time down sampling by summation (ANOVA *p*-value = 1.54 × 10^−7^). In summary, these data indicate that RT summation might enhance the quality of the obtained data for diagonal-PASEF acquisitions, especially for low-loadings and thereby confirms previous studies which indicated beneficial effects of scan summation on dia-PASEF or slice-PASEF data (12, 21).

### Retention-Time Summation Improves the Analytical Performance of Diagonal-PASEF Acquisitions, Especially for Low-inputs

**Fig. 4:**
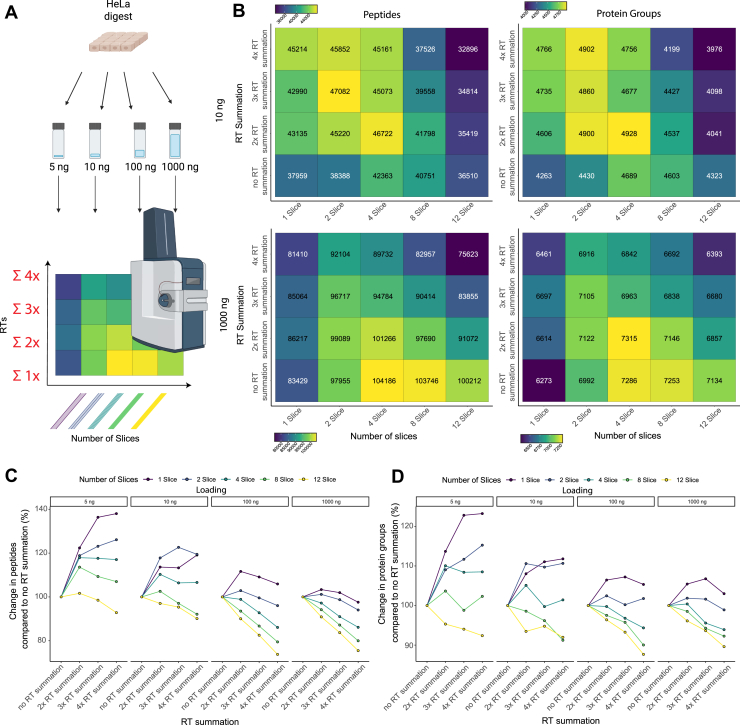
**Retention time summation improves overall identification and precision for diagonal PASEF acquisitions.***A*, schematic of experimental approach conducted to evaluate the performance of RT summation on a set of diagonal-PASEF acquisitions across different tested loadings on the timsTOF HT. *B*, results from systematic testing of the retention time summation on all diagonal-PASEF methods for 10 ng (*top*) or 1000 ng (*bottom*) of loading. Average peptide (*left)* and protein group (*right*) identification across four replicates are shown for each tested diagonal-PASEF method (x-axis) against the utilized retention time summation value (y-axis). Average identifications are indicated within the tiles. *C-D*, change in average number of peptides (*A*) or protein groups (*B*) for each tested RT summation value compared to if no summation was applied for each tested diagonal PASEF method and loading. Each datapoint represents the relative change on the average identified number of the indicated analyte across all four replicates. Panel A was generated with Biorender.com.

Given the advantage for scan summation on the signal-to-noise ratio, we next sought to evaluate the performance of diagonal-PASEF in combination with RT summation in a systematic approach. For this we injected a HeLa digest at different loading amounts on our timsTOF HT mass spectrometer ranging from 5 ng to 1000 ng of net input material per acquisition using diagonal-PASEF methods consisting of 1, 2, 4, eight or 12 slices in quadruplicates (Fig. 4*A*, Supplemental Fig. S4). We chose this slices-range to cover a wide range of DPPP and because these methods had the highest quantitative precision while allowing for a robust number of identifications in previous experiments. To initially determine the optimal RT summation for each diagonal-PASEF method and loading, we first analyzed each of the diagonal-PASEF acquisitions with RTsum set to 1 (no summation), 2, 3, or 4 for 10 and 1000 ng sample loading (Fig. 4*B*). We observed that for 10 ng of loading the method with the highest peptide identifications (47,082) was achieved with the 2-slice method subjected to 3× RT summation while most protein identifications were achieved with the 4-slice method subjected to 2× RT summation. At 1000 ng of loading the 4-slice method subjected to a 2× RT summation yielded most protein group identifications. These results confirm our previous analyses and provide supporting evidence that retention time summation might be beneficial for diagonal-PASEF acquisitions. We therefore next investigated the average protein group and peptide identifications for each of these slice-RTsum combinations as well as the average DPPP that the acquisitions would yield post RT summation to systematize the analysis (Supplemental Fig. S4). As expected, RTsum enhanced the protein group and peptide identifications strongly for diagonal-PASEF methods consisting of 1, 2 or 4 slices across all tested loadings (Fig. 4, *C* and *D* and Supplemental Fig. S4). We identified the strongest increase in the protein group and peptide identifications, especially at low loadings (<10 ng) where any tested RT summation yielded higher peptide and protein identifications when using a diagonal-PASEF method with 1, two or four slices. For example, at 5 ng of loading an RTsum value of four increased the protein group identifications by 23.2% and the peptide identifications by 38.0% compared to if no RTsum was applied for a 1-slice diagonal-PASEF method. RTsum-driven identification enhancements were observed at all loadings, but their extent faded with enhanced loading. For example, at 10 ng and 100 ng of loading applying an RTsum of four increased the peptide identifications by 19.1% and 5.9% respectively compared to no RTsum for a 1-slice diagonal-PASEF method. Interestingly, we noticed that RT summation generally proved to be detrimental for peptide or protein group identifications if applied to a diagonal-PASEF composed of eight or 12 slices **(**Figs. 4, *C* and *D* and Supplemental Fig. S4). For example, applying an RTsum of two on a diagonal-PASEF method with 12-slices reduced the peptide identifications across all tested loadings (5 ng: −1.1%, 10 ng: −3.0%, 100 ng: −10.0%, 1000 ng: −10.3%). We reasoned that RT summation is incompatible with the diagonal-PASEF method if it already sampled the chromatographic peak between 3 and 5 times. To ensure that the RT summation would not alter the quantitative precision of the diagonal-PASEF methods, we next investigated the median CVs for all diagonal-PASEF methods and RTsum values (Supplemental Fig. S5). As expected, RT summation did not systematically enhance the median CV values for any tested number of slices or loading on the peptide or protein group level. Based on these observations, we decided to use the following RT summation values for our tested diagonal-PASEF methods across all loadings: 1-slice: 4× RTsum; 2-slice: 3× RTsum; 4-slice: 2× RTsum; 8-slice: 1× RTsum; 12-slice: 1× RTsum. Finally,to validate that the RT summation concept is also applicable for diagonal-PASEF data acquired in other studies, we next obtained data from a recently published study (Skowronek *et al* Nature Protocols 2025) (18). In this study, the authors developed 2- and 4-slice diagonal-PASEF methods that were applied in 30 SPD methods on K562 cell line digests. Strikingly, RTsum enhanced the peptide and protein group identification of the 2-slice equal-sized diagonal-PASEF method and even the proposed 4-slice diagonal-PASEF method that showed variable slice-widths (Supplemental Fig. S6). On the protein level RT summation enhanced the identifications by up to 11.1% (RTsum = 4) for the 2-slice method and up to 7.1% (RTsum = 3) for the 4-slice method. These identifications thereby outcompete the published results from the study where the data was obtained and provide further evidence that RTsum is indeed valuable for the analysis of diagonal-PASEF data.

### Diagonal-PASEF with RT Summation Yields Equal to Higher Identifications at Higher Precision than Dia-PASEF

**Fig. 5:**
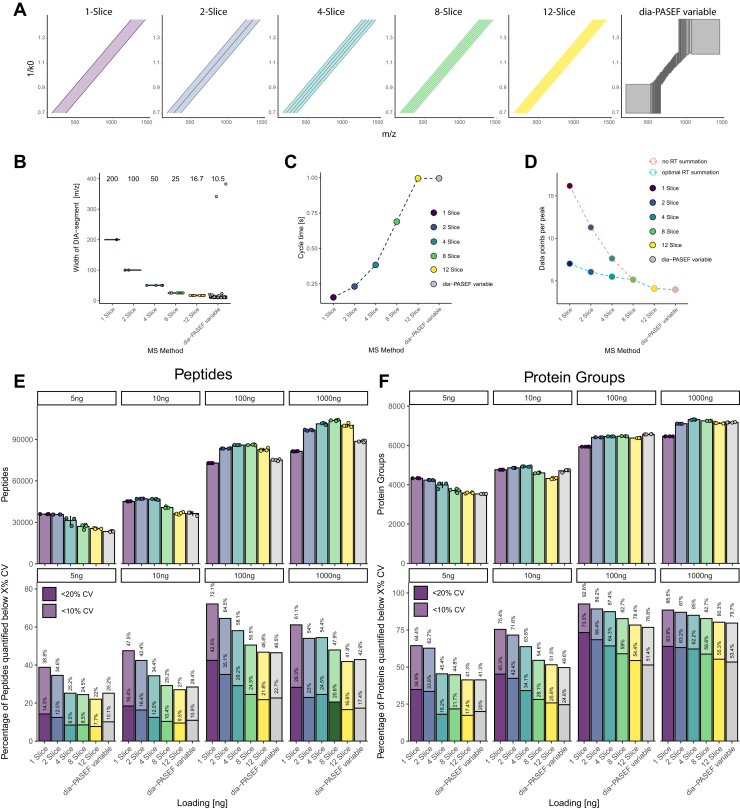
**Diagonal-PASEF achieves higher overall identification and precision compared to dia PASEF.***A*, schematic of diagonal-PASEF and dia-PASEF methods used to evaluate the performance of diagonal-PASEF on the timsTOF HT. *B*, width of the DIA-segments of each applied method for tested methods from *A*. Each *datapoint* indicates an individual slice or DIA-segment from the diagonal-PASEF or dia-PASEF method. *Boxplots* have been added behind the data points to visualize the distribution of the DIA segment width. The median of the segments is depicted a the *top* of the plot. *C*, cycle time of tested methods for loading ramp experiment depicted in *A*. *D*, data points per peak of tested methods for loading ramp experiment pre (*red*) or post (*blue*) retention time summation. For the RT summation, the ideal value has been applied as follows: 1-slice: 4xRTs, two slice: 3xRTs, 4-slice: 2xRTs, 8-slice: 1xRTs, 12-slice: 1xRTs. *E–F*, results of loading ramp. Top: Average peptide (*E*, *left*) or protein groups (*F*, *right*) identifications obtained for all tested methods across the indicated loadings. *Error bars* indicate standard deviation of mean. Each *datapoint* indicates an individual acquisition (n = 4). *Bottom*: Percentage of peptides (*E, left*) or protein groups (*F, right*) quantified below 10% or 20% coefficient of variation (CV) against overall identifications. Labels show analytes quantified below 10% or 20% CV. Each bar represents the result of one search. For panels *D* and *E*, the optimal retention time summation was applied to all diagonal-PASEF acquisitions as follows: 1-Slice: 4xRTs, 2-slice: 3xRTs, 4-slice: 2xRTs, 8-slice: 1xRTs, 12-slice: 1xRTs.

Next, we aimed at comparing the performance of diagonal-PASEF driven by RT summation against classic dia-PASEF acquisition. For this, we acquired a dia-PASEF method composed of windows with variable widths (termed dia-PASEF variable) that we computed using an in-house software and optimized to maximize protein group identifications alongside all diagonal-PASEF acquisitions (Fig. 5, *A* and *B*). We validated the performance of the generated dia-PASEF method by comparing it to a method that we generated using the “py-diAID” tool which is commonly used in the proteomics community for low to high input amounts (Supplemental Fig. S7) (19). We designed the dia-PASEF method to consist of exactly 12 PASEF ramps such that these methods would have the same cycle time and DPPP compared to the diagonal-PASEF method consisting of 12 slices (12 PASEF ramps, Fig. 4, *B* and *C*). On average, the tested diagonal-PASEF methods with RT summation yielded higher peptide and protein group identifications compared to the dia-PASEF control method across all tested loadings (Fig. 4, *E* and *F*). Diagonal-PASEF methods composed of <4 slices generally provided more peptide and protein group identifications at lower loadings. For example, at 5 ng of loading the 1-slice diagonal-PASEF method yielded 38.6% more peptides than the dia-PASEF method. On the protein group level, the same method yielded 17.0% more protein groups than the dia-PASEF method. In contrast, at the higher tested loadings of 100 and 1000 ng, the best diagonal-PASEF method generally had four or more slices. At the highest tested loading, the best performing methods on the protein level were the 4-slice and 8-slice methods which achieved 1.9% and 1.1% more proteins than the dia-PASEF control. These methods also yielded generally more peptides than dia-PASEF, albeit for some methods gains in peptide identifications did not directly yield more identified protein groups. As an example, the 12-slice diagonal-PASEF method yielded 0.4% less protein groups but 14.1% more peptides than the highly optimized dia-PASEF variable method. We hypothesized that the 12-slice method would have an enhanced sequence coverage to the detriment of the overall number of protein identifications. Indeed, we could confirm this hypothesis by evaluating the average number per peptides detected per protein group across all investigated methods (Supplemental Fig. 8). In summary, these data indicated that diagonal-PASEF are competitive against dia-PASEF method across all tested loadings on the timsTOF HT at overall protein and peptide identifications.

Next, we investigated the quantitative precision of the tested dia-PASEF and diagonal-PASEF methodologies. For this we computed the protein groups or peptides quantified below 20% or 10% coefficient of variation (CV, Supplemental Fig. S9*A*) and analyzed the analytes quantified below a certain CV threshold relative to the overall identifications obtained (Fig. 5, *E* and *F*). All tested diagonal-PASEF methods provided very high quantitative precision. In general, we noticed that the relative quantitative precision was reduced with an increasing number of diagonal-PASEF slices across the tested methods. For example, at 100 ng of loading 72.1% of all peptides were quantified below 20% CV using a 1-slice method, whereas with the 12-slice method, only 46.5% of all peptides were quantified below 20% CV (Fig. 5*E*). Despite this effect, the tested diagonal-PASEF had a notably higher quantitative precision compared to the investigated dia-PASEF method. At 5 ng of loading, the average tested diagonal-PASEF method quantified 29.0% of all peptides and 51.7% of all proteins with less than 20% CV, while the dia-PASEF method quantified only 25.2% or 41.3% of all peptides or proteins, respectively, with less than 20% CV. The same pattern was observed for all other tested loadings. For example, on the peptide level, the average diagonal-PASEF method quantified 29.2%, 36.1%, or 58.4% of all peptides below 20% CV at a loading of 5, 10, or 100 ng, while the dia-PASEF method quantified only 25.2% 28.4% or 46.5% of all peptides below 20% CV. These trends also hold up when quantifying the absolute number of analytes below 10% or 20% CV whereby diagonal-PASEF methods generally outcompeted the dia-PASEF methodology regardless of the utilized loading (Supplemental Fig. S9*A*). In summary, these data indicate that diagonal-PASEF methods not only provide higher overall identification rates than dia-PASEF methods but also allow for a better quantification of the detected analytes.

To dissect whether the higher number of quantified proteins or peptides arose due to overall higher identification rates of diagonal-PASEF, we next looked at the run-to-run correlation of all replicate injections for all methods and loadings (Supplemental Fig. S9, *B*–*G*). As expected, we identified a higher average run-to-run correlation for the diagonal-PASEF compared to the dia-PASEF acquisitions at all tested loadings. All tested diagonal-PASEF methods had an average run-to-run correlation on the peptide level as expressed by the Spearman-based rank correlation rho value of 0.902, 0.937, 0.972, and 0.963 at 5, 10, 100, and 1000 ng of loading, respectively. In comparison, the dia-PASEF method only achieved a correlation rho value of 0.891, 0.913, 0.951, and 0.943 at 5, 10, 100, and 1000 ng of loading, respectively (Supplemental Fig. S9*C*). We generally observed all methods to achieve a better run-to-run correlation at higher loadings, with the best correlation being obtained at 100 ng of loading (Supplemental Fig. S9*D*). These results were also confirmed on the protein group level, although the detected differences were smaller, consistent with the observed trends for proteins and peptide CV in Figure 2 (Supplemental Fig. S9, *E*–*G*). The largest difference in the run-to-run correlation was observed at 5 ng of loading. Here, the 1-slice diagonal-PASEF method had the best run-to-run correlation with a Spearman rho of 0.969, while that of the dia-PASEF method was only 0.924. Interestingly, when looking into individual run-to-run correlations, we noticed that diagonal-PASEF appears to be more suited to precisely quantify especially low-abundant proteins (Supplemental Fig. S10, *A* and *B*). To dissect the quantitative precision of individual analytes across instrument methods, we assessed the correlation between various diagonal-PASEF configurations and the dia-PASEF reference (Supplemental Fig. S11). All tested diagonal-PASEF methods correlated similarly well with the dia-PASEF control at all tested loadings. At 5 ng of loading, the 1- and 2-slice methods achieved the highest correlation (rho = 0.93), while at 1000 ng of loading, the 8- and 12-slice methods reached peak correlation values (rho = 0.97). In agreement with this data, we noticed that there was a high-degree of overlapping analytes between the tested diagonal-PASEF methods and the dia-PASEF control across all tested loadings (Supplemental Fig. S12).

Taken together, these findings indicate that while diagonal-PASEF outperforms dia-PASEF in terms of overall identifications, quantitative precision and run-to-run correlation, both acquisitions yield highly comparable data.

### Diagonal-PASEF Results in a Greater Number of Truly Differentially Abundant Proteins in Controlled Quantitative Experiments Compared to Dia-PASEF

**Fig. 6:**
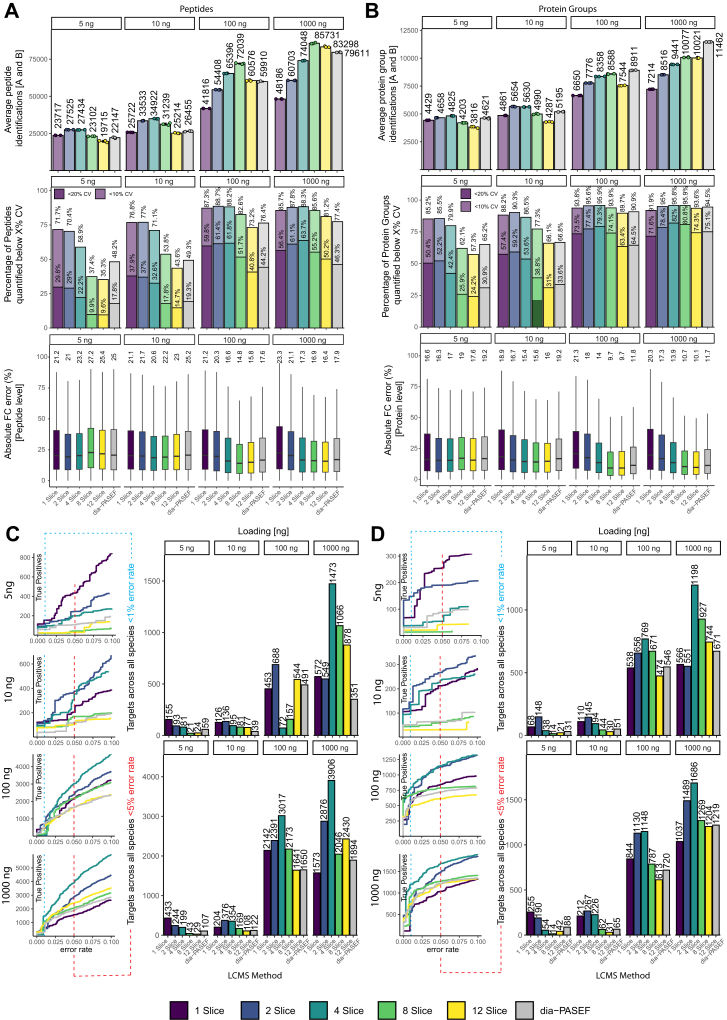
**Diagonal-PASEF and retention time summation outperform dia-PASEF acquisitions in controlled quantitative experiments.***A–B*, results from CQE loading ramp. *Top*: Average peptide (*A*) or protein group (*B*) identifications across samples *A* and *B* combined for all indicated diagonal-PASEF or dia-PASEF methods for the indicated loadings. Data points indicate individual replicates (n = 3). Mean and standard deviation of mean shown. Average identifications are depicted above error bars. *Middle*: Percentage of identifications below 20% CV (faint) or 10% CV (bold) as percentage of overall identifications averaged from samples *A* and *B* on peptide (*A*) or protein group level (*B*). Percentage values above bars indicate the height of bars. *Bottom*: Absolute fold change (FC) error (%) on peptide (*A*) or protein group (*B*) level for indicated methods and tested loadings. Median absolute fold change errors are indicated above the boxplots. *Boxplots* indicate the inter-quartile range (IQR) from the lower quartile to the *upper quartile*. *Central line* indicates the median value of the population, and whiskers indicate the 1.5 × IQR. Median CV is indicated above the plot. Outliers are not shown to aid the visual interpretation of the data. *C*–*D*: *Left*: True positive (TP) identifications over the error rate of candidates shown for diagonal-PASEF and dia-PASEF methods on peptide (*C*) or protein group (*D*) level. *Dashed vertical line* indicates an error rate of 0.01 (*blue*) or 0.05 (*red*) at which the number of true positives was read out. *Right*: Number of candidates with error rate <0.01 (*top right*) or < 0.05 (*bottom right*) for the indicated method and loading. Values above bars indicate the number of true positives below the indicated threshold (height of the bar).

Given that the tested diagonal-PASEF methods in conjunction with RTsum yielded more peptide and protein identifications as well as higher quantitative precision compared to dia-PASEF, we next evaluated the methods in more realistic scenarios. We and others have previously shown that controlled quantitative experiments (CQEs) are a suitable tool in assessing key metrics of analytical LCMS methods through the derivation of a ground truth (7). Therefore, we set out and prepared a CQE in which we combined four different species (*H. sapiens*, *C. elegans*, *S. cerevisiae*, *E. Coli*) in different ratios (materials & methods) and prepared two distinct samples termed “A” and “B” which would yield predefined fold changes when measured together (Supplemental Fig. S13*A*). We then acquired these samples at 5, 10, 100 and 1000 ng of loading on the timsTOF HT with a 17-min analytical gradient. We compared diagonal-PASEF methods consisting of 1, 2, 4, eight or 12 slices which were subjected to RT summation against the dia-PASEF method. The resulting data were acquired in triplicates, and we investigated several key-parameters which we have previously shown to contribute to the analytical performance of LCMS methods in CQEs (7). These include i) an identification metric for which we summarized the peptides or protein groups from samples A & B, ii) a precision metric for which we computed the CV and number of analytes below 20% and 10% CV and iii) an accuracy metric for which we computed the median fold change error (Fig. 6, *A* and *B*). First, we investigated the data on the peptide level (Fig. 6, *A* and *C*, Supplemental Fig. S13, *C* and *E*, Supplemental Fig. S14, *A* and *C*). The 2-slice diagonal-PASEF method had the overall highest peptide identifications at a tested loading of 5, while at 100 ng and 1000 ng of loading the 8-slice diagonal-PASEF method achieved higher peptide identifications (Fig. 6*A*, Supplemental Fig. S13*B*). At the highest tested loading of 1000 ng, the 8-slice diagonal-PASEF method achieved 7.7% more peptide identifications than the dia-PASEF method. These data confirm previous findings and indicate that diagonal-PASEF methods with a larger number of slices are generally more suited for larger loadings than methods with smaller number of slices (Fig. 5). Next, we evaluated the quantitative precision of the tested LCMS methods. The one and 2-slice diagonal-PASEF methods had the highest overall relative quantitative precision across all tested loadings on the peptide level (Fig. 6*A*, Supplemental Fig. S13*C*). At 5 ng of loading, the 1-slice diagonal-PASEF method quantified 71.7% and 29.8% of all analytes with a CV below 20% and 10% respectively, whereas the dia-PASEF method only quantified 48.2% and 17.8% of all analytes with a CV below 20% and 10% respectively. The 12-slice diagonal-PASEF method had the overall worst quantitative precision at the lowest tested loading. At the highest tested loading of 1000 ng, the 4-slice method quantified 88.3% and 63.7% of all analytes below 20% and 10% CV whereas the dia-PASEF method only quantified 77.4% and 46.3% of all analytes below 20% or 10% CV respectively. This data indicates that the diagonal-PASEF method provides high peptide identifications at a higher quantitative precision compared to the tested dia-PASEF method at all investigated loadings. Finally, we evaluated the absolute fold-change error that we computed as percentage of the deviation between the empirically determined fold change by the analytical method and the expected fold change based on the experimental design (materials & methods, Fig. 6*A*, Supplemental Fig. S13*E*). At loadings of 100 and 1000 ng we identified the 8- and 12-slice diagonal-PASEF method to achieve the best quantitative accuracy with an average median fold-change error of 15.9% and 16.1%, respectively, compared to 22.2%, 20.7%, 16.9%, and 17.8%for the 1-, 2-, and 4-slice and dia-PASEF methods respectively. We noticed that for lower loading of 5, and 10 ng, methods with 4-slices or less appeared to have an overall best quantitative accuracy compared to the 8- and 12-slice and the dia-PASEF method. Despite these differences, all tested methods achieved a comparable fold-change error indicating that all methods have a similar quantitative accuracy. Taken together, these data indicate that on the peptide level diagonal-PASEF methods achieve higher identifications and better quantitative precision together with a comparable quantitative accuracy as the dia-PASEF method.

An important aim of proteomics projects is to detect differentially abundant proteins or peptides which arise from, for example, statistical testing of biological conditions. Therefore, we also investigated the number of true candidates below 1% and 5% error rate for all tested loadings and acquisition methods to provide a more complete evaluation of the CQE (Fig. 6*C*). For this, we performed pair-wise testing of all peptide identifications between samples A and B, assessed whether the analytes followed the projected fold-change trajectory and counted the number of correctly quantified true candidates below an error rate of 1% and 5% (material & methods). Diagonal-PASEF achieved more candidates on the peptide level across all tested loadings compared to dia-PASEF at both investigated error rates. At 5 ng of loading the 1-slice method allowed to identify 155 and 433 candidates at an error rate of 1% or 5%, respectively, which was 2.62-fold or 4.4-fold more targets than the dia-PASEF method achieved at this loading. At 1000 ng of loading the 4-slice diagonal-PASEF identified the most candidates at 1% and 5% error rate with 1473 and 3906 true positive identifications respectively, which was 4.19-fold and 2.06-fold more than the dia-PASEF method achieved. We confirmed these findings by computing the partial area under the curve (pAUC) for the true positives versus candidate list plots below an error rate of 5% (Supplemental Fig. S14*C*). These data indicate that diagonal-PASEF can yield more target identifications than dia-PASEF at all tested loadings and might thus be a competitive alternative to dia-PASEF for peptide-level interrogation of datasets.

Next, we repeated these analyses for all tested diagonal-PASEF and dia-PASEF methods on the protein group level (Fig. 6, *B* and *D*; Supplemental Fig. S13, *D* and *F*, Supplemental Fig. S14, *B* and *D*). On the identification level, we found the dia-PASEF variable method to achieve the overall highest identifications at 100 and 1000 ng of loading in the CQE indicating that dia-PASEF is indeed very competitive in measuring complex samples such as CQEs. Notably, at a loading of 5 and 10 ng, the 2- and the 4-slice diagonal-PASEF method achieved more protein group identifications. When investigating the quantitative precision, we again identified the 1- and 2-slice diagonal-PASEF method to quantify most analytes below 20% or 10%, especially at loadings below 100 ng, similar to the data previously obtained for the peptide level (Fig. 6*A*). At a loading of 1000 ng, however, the 4- and 8-slice methods quantified most analytes below 20% and 10% CV, respectively. In agreement with this, the median fold-change error was also observed to be similar for all tested methods. As on the peptide-level data the 8- and the 12-slice diagonal-PASEF method achieved the lowest average median fold change error of 12% and 11.9%, respectively for loadings of 10, 100 and 1000 ng, whereas the dia-PASEF and the 1, 2, and 4-slice diagonal-PASEF method had a median fold change error of 14.2%, 14.4%, 17.3% and 20.2% respectively. Only at 5 ng loading, the method with the highest quantitative accuracy was the 1-slice and 2-slice methods. Taken together, these data indicate that while dia-PASEF achieves most protein group identifications, the diagonal-PASEF methods generally present with a higher quantitative precision.

Next, we computed the true candidates retained below an error rate of 1% and 5% (Fig. 6*D*) on the protein group level. Similar to the peptide-level data at the lowest tested loading of 5 and 10 ng, 148 and 145 true candidates were identified using the 2-slice method at 1% error rate, which is 4.77-fold and 2.84-fold more than the dia-PASEF method. At an error rate of 5%, most candidates were achieved using the 1-slice method at 5 ng (255) or the 2-slice method at 10 ng (267). At higher loadings, the 4-slice diagonal-PASEF method outcompeted all other diagonal-PASEF methods and achieved 769 and 1198 candidate identifications at 100 and 1000 ng of loading, respectively, at 1% error rate, which was 1.4-fold or 1.78-fold more than the dia-PASEF control, respectively. At an error rate of 5%, the same relative performance was obtained, and most candidates were again identified using the 4-slice method with 1148 and 1686 correctly identified targets at 100 ng or 1000 ng of loading. In summary, these data indicate that at the protein level, diagonal-PASEF methodologies outcompete dia-PASEF methods at the tested loading range, but at higher loadings, dia-PASEF is very competitive against diagonal-PASEF.

## Discussion

Two pioneering research groups have recently demonstrated diagonal-PASEF method architectures which are commonly referred to as midia-PASEF or synchro-PASEF on timsTOF instruments. While these attempts have created interest in these novel methods, their application in real-world scenarios has so far not been realized, which can at least partially be attributed to a lack of available algorithm solutions that analyze this type of data. In this study, we present a novel data analysis strategy for fast-cycling diagonal-PASEF which utilizes retention time-based down-sampling which leads to the sum of adjacent MS1 and MS2 scans. This data analysis strategy has been implemented since Spectronaut 19, and we tested this specifically with Spectronaut 20 and used it to comprehensively optimize several diagonal-PASEF method parameters. In our systematic evaluation, we report that RT summation significantly improves the peptide and protein group identifications for fast-cycling diagonal-PASEF methods. In the human cell line HeLa, RTsum-enhanced diagonal-PASEF resulted in higher peptide identifications and protein identifications compared to dia-PASEF. Importantly, the precision at both the peptide and protein levels was consistently higher with diagonal-PASEF than with dia-PASEF. In a controlled quantitative experiment, diagonal-PASEF yielded similar accuracy in fold-change measurements as dia-PASEF but generally identified a higher number of true positive peptide candidates at an error rate of 1% and 5% (based on the *t* test results), compared to traditional dia-PASEF. Strikingly, we also observed diagonal-PASEF to achieve a higher number of true positive protein group candidates at an error rate of 1% or 5% at the tested loadings. This indicates that diagonal-PASEF in conjunction with RT summation can be highly competitive in identifying differentially expressed proteins and peptides.

We found that diagonal-PASEF can efficiently cover the main ion distribution of the TIMS elution with as few as one to four slices. Consequently, these methods can have very short cycle times (∼0.05–0.5 s), allowing for oversampling of chromatographic peaks that typically elute over several seconds (∼3–9 s). To optimally analyze oversampled data, we summed corresponding consecutive MS1 and MS2 scans over the RT dimension. Methods with fewer slices, i.e., with shorter cycle times, allow for a higher degree of down-sampling compared to methods with more slices. Methods with the highest number of slices (8, 12) were incompatible with down-sampling, as the cycle time of the resulting method would not sufficiently sample the chromatographic peaks. When optimizing diagonal-PASEF, we observed that for lower sample amounts (5 and 10 ng), methods with fewer slices were superior to those with higher slice numbers when combined with RT summation (Fig. 4). We reasoned that this enhancement of the identifications could be due to the observed increase in signal-to-noise ratio for methods, where retention time down-sampling is applied (Fig. 3). Improvements in the signal-to-noise ratio likely led to better identification and higher precision for low sample amounts, compared to methods without retention time down-sampling. We reason that in such cases, many signals might be close to the noise floor, making the signal-to-noise ratio a limiting factor for the search engine to translate spectra into peptide identifications. While the general concept of scan summation is not novel as it has been applied on dia-PASEF (12) or slice-PASEF (21) acquisitions recently, we provide here for the first time a systematic assessment of RT summation on a range of diagonal-PASEF methods. When applying RT summation to existing diagonal-PASEF data (18) we could further demonstrate that the scan-summation concept can enhance the identification and quality of existing fast-scanning acquisitions. For HeLa, we observed that diagonal-PASEF generally achieved higher peptide identifications than dia-PASEF when combined with RT summation. At the protein level, identifications were similar for both methods although diagonal-PASEF achieved slightly more identifications at all loadings except 100 ng, leading to better sequence coverage of the identified proteins and, consequently, more information on peptide-specific traits such as post-translational modifications (PTMs), isoforms, and proteoforms. Strikingly, we found the quantitative precision of peptides and proteins to be consistently higher for diagonal-PASEF compared to dia-PASEF, confirming recent data from another study (18). These findings render diagonal-PASEF particularly well-suited for studies aiming at peptide-level data, such as PTMs, peptidomics-based approaches, and immunopeptidomics. To evaluate the performance of diagonal-PASEF supported by RT summation for detecting differentially abundant analytes, we performed a controlled quantitative experiment and benchmarked it against dia-PASEF. We found peptide identifications to be higher across all tested sample loading amounts for the best diagonal-PASEF compared to the dia-PASEF method. In contrast to the observations from the single human proteome experiments using HeLa, at the highest sample loading, dia-PASEF yielded the most protein identifications. CQEs rely on mixing multiple proteomes and are thus significantly more complex. Currently, this higher complexity seems to be handled more efficiently by the dia-PASEF data analysis pipeline. However, CQEs do not reflect the reality for most proteomics projects, which commonly comprise only one organism of interest. The variable dia-PASEF method and the diagonal-PASEF method with more narrow slices (8-slice, 12-slice) generally had a similar accuracy. Interestingly, we found the 2-slice diagonal-PASEF method to achieve the lowest accuracy at the high loadings, possibly due to the high number of interferences caused by the wide quadrupole isolation window. When performing statistical testing for differential abundance, accuracy, precision, and identifications are relevant. We found diagonal-PASEF to yield more true positive candidates at both peptides and protein level that are statistically differentially abundant for all investigated sample loading amounts compared to dia-PASEF below an error rate of 1%. The increase in statistical power is explained by the increase in precision while maintaining accuracy. As with the HeLa peptide loading ramp, for low peptide loading amounts, diagonal-PASEF with few slices performed better, and at higher peptide loads, the method with more slices proved superior. Finally, dia-PASEF candidate recovery performance was best at the loading of 1000 ng when an error rate of 5% was applied, correlating with the highest identification of dia-PASEF at this loading. While results from this study indicate that diagonal-PASEF is superior compared to dia-PASEF in CQE scenarios, follow-up experiments should be conducted whereby diagonal-PASEF is compared to a range of dia-PASEF methods that are characterized by different DIA geometries and cycle times.

Future improvements for diagonal-PASEF could include acquisitions with very narrow slices. In this study, we kept the overall width of the diagonal-PASEF method constant at 200 m/z and the width of the slices equal. Other studies have shown that modulating this parameter could enhance the performance of diagonal-PASEF (18). Therefore, in a more systematic assessment, the width of the diagonal-PASEF method could be adjusted against parameters such as the ramping time of the TIMS cells or the number of diagonal-PASEF slices to identify the optimal configuration. Additionally, the instrument control software for ion mobility and quadrupole scanning dynamics is being actively developed to enable new acquisition modes in the future, including variable slice methods that have recently been proposed (18). On the data analysis side, additional algorithms like the proposed precursor slicing (22) and fingerprint midia-PASEF type of analyses (23) could increase both identification and quantification performance. In this study, we also only benchmarked down-sampling by scan-summation, but in alternative approaches, down-sampling can also be achieved through averaging scans. The systematic evaluation of such down-sampling strategies could further enhance the utility of diagonal-PASEF beyond what has been shown in this report.

Despite being a nascent technology, diagonal-PASEF already is on par with dia-PASEF in key metrics such as peptide identification and quantification. This is of particular interest for workflows focused on PTMs, peptidomics, and immunopeptidomics, where higher-quality peptide-level data can dramatically improve results. We expect this novel acquisition method to become the preferred acquisition methodology in the near future.

## Data Availability

The raw MS data, the spectral libraries and the quantitative data tables have been deposited to the ProteomeXchange Consortium via the MassIVE partner repository with the dataset identifier MSV000099586. The Saved projects from Spectronaut can be viewed with the Spectronaut Viewer (www.biognosys.com/spectronaut-viewer).

To view the dataset's web page (including title, description, and metadata), log in at the upper right corner of this page.

If you are already logged in to another MassIVE account, then you can first log out by clicking the “Logout” link in the upper menu.

Username for web access: MSV000099586_reviewer

To view the dataset's files, log in to the MassIVE FTP server with this URL: ftp://MSV000099586@massive-ftp.ucsd.edu

We recommend using a dedicated FTP client to do this, though most web browsers will also support basic FTP access.

Username for FTP access: MSV000099586

Password: 2Y0p4iqZqRCeuAlW

## In Memoriam

We would like to dedicate this work to Lukas Reiter, who sadly passed away during the revision of this manuscript. His contributions to this study and to the field of proteomics were invaluable, and he will be deeply missed.

## Supplemental Data

This article contains supplemental data (22, 30).

## Conflict of Interest

The authors declare the following financial interests/personal relationships which may be considered as potential competing interests:

The authors B. S., C. B., L. RA., L. R., M. P., O. M. B., R. B., S. S. and T.G. are full-time employees of Biognosys AG (Zurich, Switzerland). Spectronaut is a trademark of Biognosys AG. D. T., E. C., J. K., S. K. S. and S. W. are employees of Bruker Daltonics GmbH & Co KG, manufacturer of the instrumentation used in this work.
