# Supplementary material for: Enhanced Identifications and Quantification Through Retention Time Down-Sampling in Fast-Cycling Diagonal-PASEF Methods
Source: Mol Cell Proteomics. 2025 Dec 9;25(1):101480. doi: 10.1016/j.mcpro.2025.101480 (PMC12818240; doi:10.1016/j.mcpro.2025.101480)
Supplement: Supplementary Figures [file mmc1.pdf]

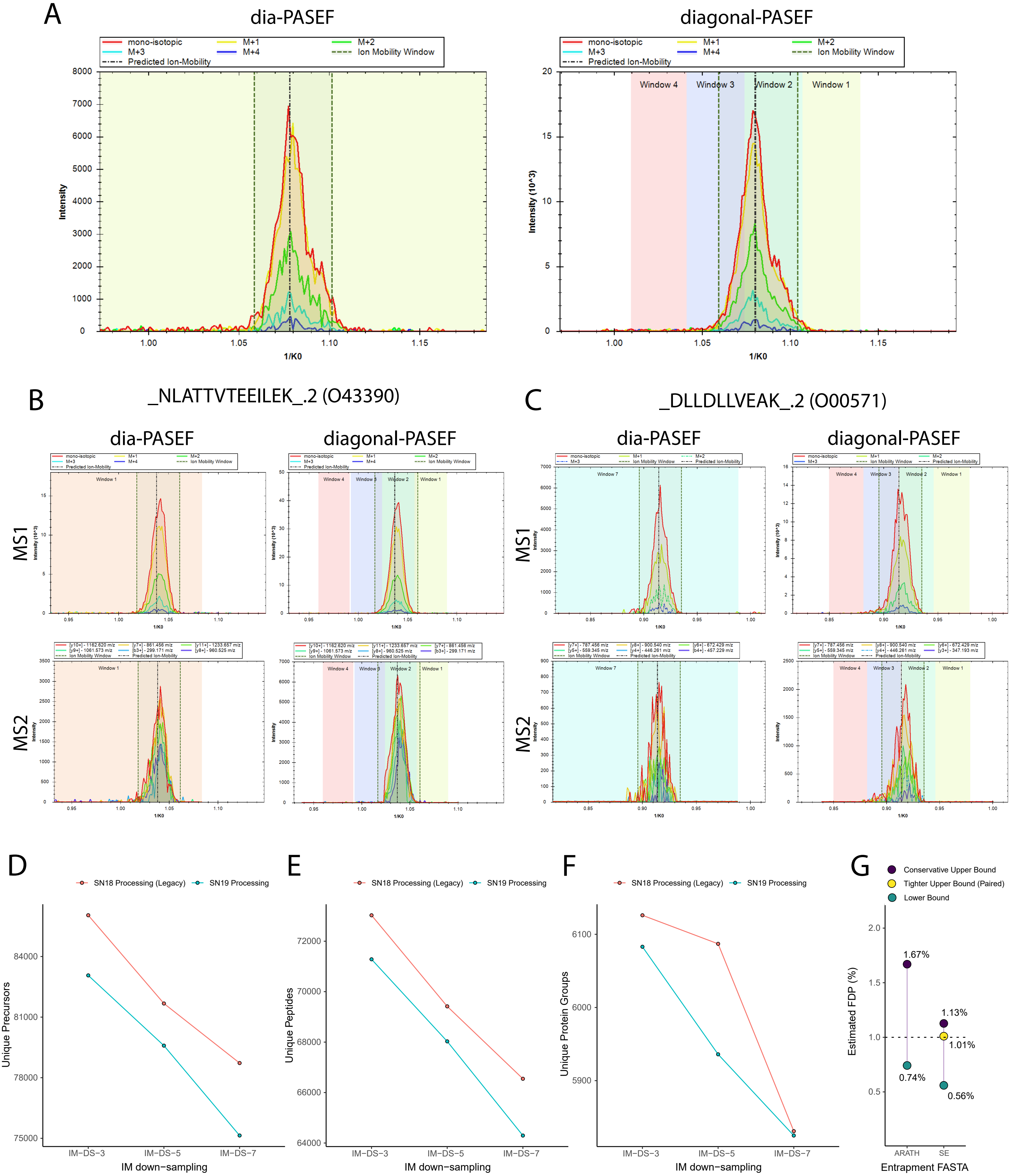
A
dia-PASEF
diagonal-PASEF
mono-isotopic
M+1
M+2
M+3
M+4
Ion Mobility Window
Predicted Ion-Mobility
Intensity
Intensity (10^3)
1/K0
Window 4
Window 3
Window 2
Window 1
B
NLATTVTEEILEK.2 (O43390)
C
DLLDLLVEAK.2 (O00571)
MS1
MS2
Window 7
D
E
F
SN18 Processing (Legacy)
SN19 Processing
Unique Precursors
Unique Peptides
Unique Protein Groups
IM down−sampling
IM−DS−3
IM−DS−5
IM−DS−7
G
Conservative Upper Bound
Tighter Upper Bound (Paired)
Lower Bound
Estimated FDP (%)
1.67%
0.74%
1.13%
1.01%
0.56%
ARATH
SE
Entrapment FASTA

Supplementary Figure 1: A: Extracted ion mobilogram (XIM) of a representative precursor "_DLYEDELVPLFEK_.2" on MS1-level from a dia-PASEF (left) or diagonal-PASEF (right) acquisition. Isotopic envelope depicted as differentially colored lines. Data-extraction range is depicted as dashed vertical line. Isolation windows governed by the acquisition method are shown as colored background. **B-C:** Extracted ion mobilogram for precursor "_NLATTVTEEILEK_.2" (**B**) and "_DLLDLLVEAK_.2" on MS1 (top) or MS2 (bottom) level from dia-PASEF (left) or diagonal-PASEF (right) acquisitions. Individual fragments are depicted as differentially colored lines. Data-extraction range is depicted as dashed vertical line. Isolation windows governed by the acquisition method are shown as colored background. **D-F:** Optimization of Spectronaut parameters "DIA pre-processing" and "Ion Mobility down-sampling" for a 100 ng HeLa acquisition using 2-slice diagonal-PASEF method at 17-minute analytical gradients on precursor (**D**), peptide (**E**) or protein group (**F**) levels. **G**: Results from FDR-validation using the entrapment method from Wen et al Nature Methods (2025) for a 100 ng HeLa acquisition using a 4-slice diagonal-PASEF method searched with Spectronaut Parameters: DIA pre-processing = "SN18 Processing (Legacy)" and IM Downsampling = 3. Entrapment FDP (False discovery proportion) was calculated using synthetic entrapment FASTA (SE) and *Arabidopsis thaliana* FASTA (ARATH). Indicated values depict computed entrapment FDP values as specified boundaries.

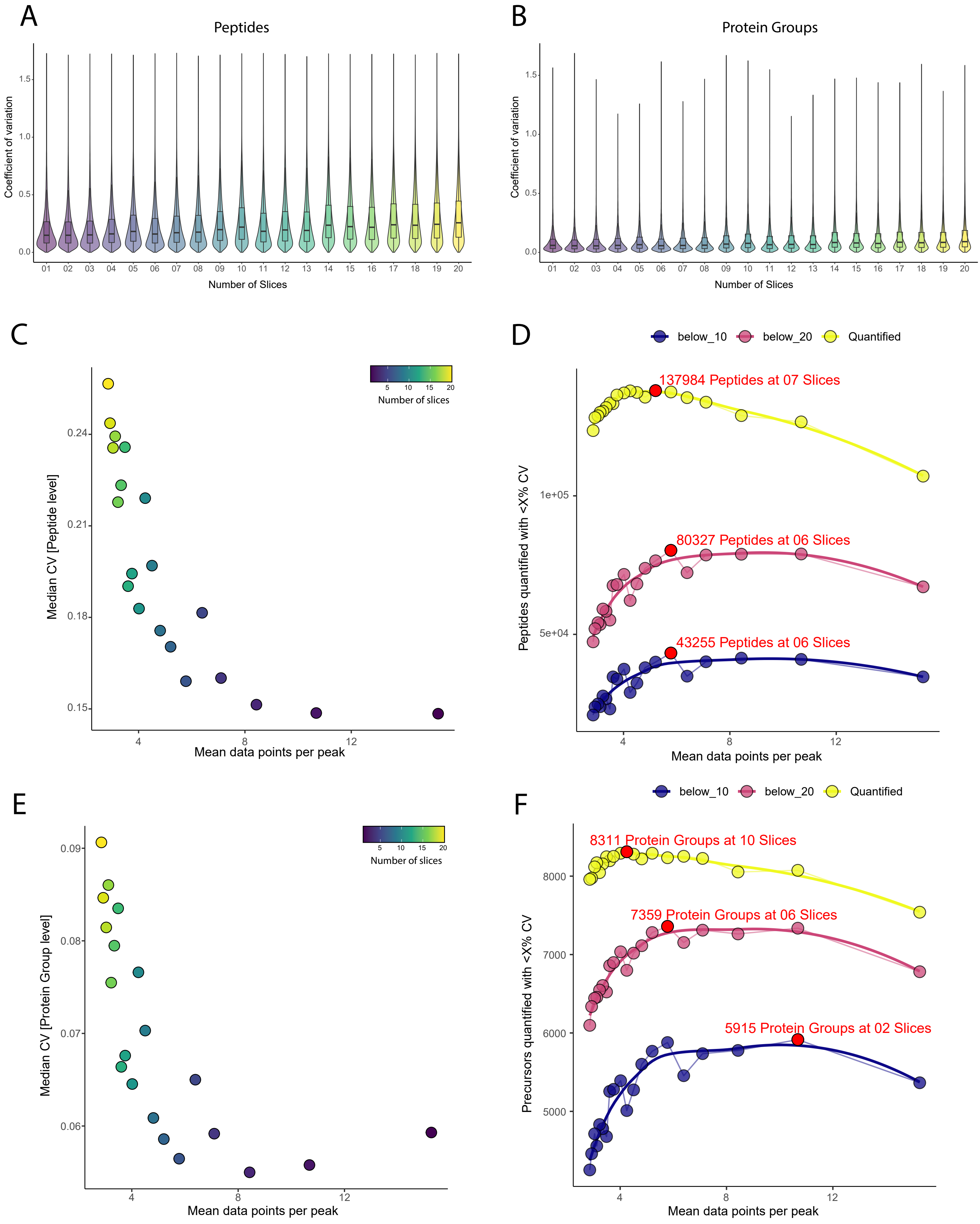
A
Peptides
Coefficient of variation
Number of Slices
B
Protein Groups
Coefficient of variation
Number of Slices
C
Number of slices
Median CV [Peptide level]
Mean data points per peak
D
below_10
below_20
Quantified
137984 Peptides at 07 Slices
80327 Peptides at 06 Slices
43255 Peptides at 06 Slices
Peptides quantified with <X% CV
Mean data points per peak
E
Number of slices
Median CV [Protein Group level]
Mean data points per peak
F
below_10
below_20
Quantified
8311 Protein Groups at 10 Slices
7359 Protein Groups at 06 Slices
5915 Protein Groups at 02 Slices
Precursors quantified with <X% CV
Mean data points per peak

Supplementary Figure 2. A-B: Coefficient of variation (CV) of all peptide (left, A) or protein group (right, B) identifications for all tested diagonal-PASEF methods. Boxplots indicate the inter-quartile range (IQR) from the lower quartile to the upper quartile. Central line indicates the median value of the population and whiskers indicate the 1.5 x IQR. Median CV is indicated above the plot. Outliers are not shown to aid the visual interpretation of the data (n = 3). **C, E**: Scatter plot of mean data points per peak against the median coefficient of variation computed on the peptide (C) or protein group (E) level. Each data point depicts an individual diagonal-PASEF method averaged from three replicates (n = 3). **D, F**: Mean data points per peak of each method against the peptides (D) or protein groups (F) quantified, quantified below 20% CV or 10% CV. The best performing method is indicated in red for each line. Each data point represents an individual method average from three replicates (n = 3).

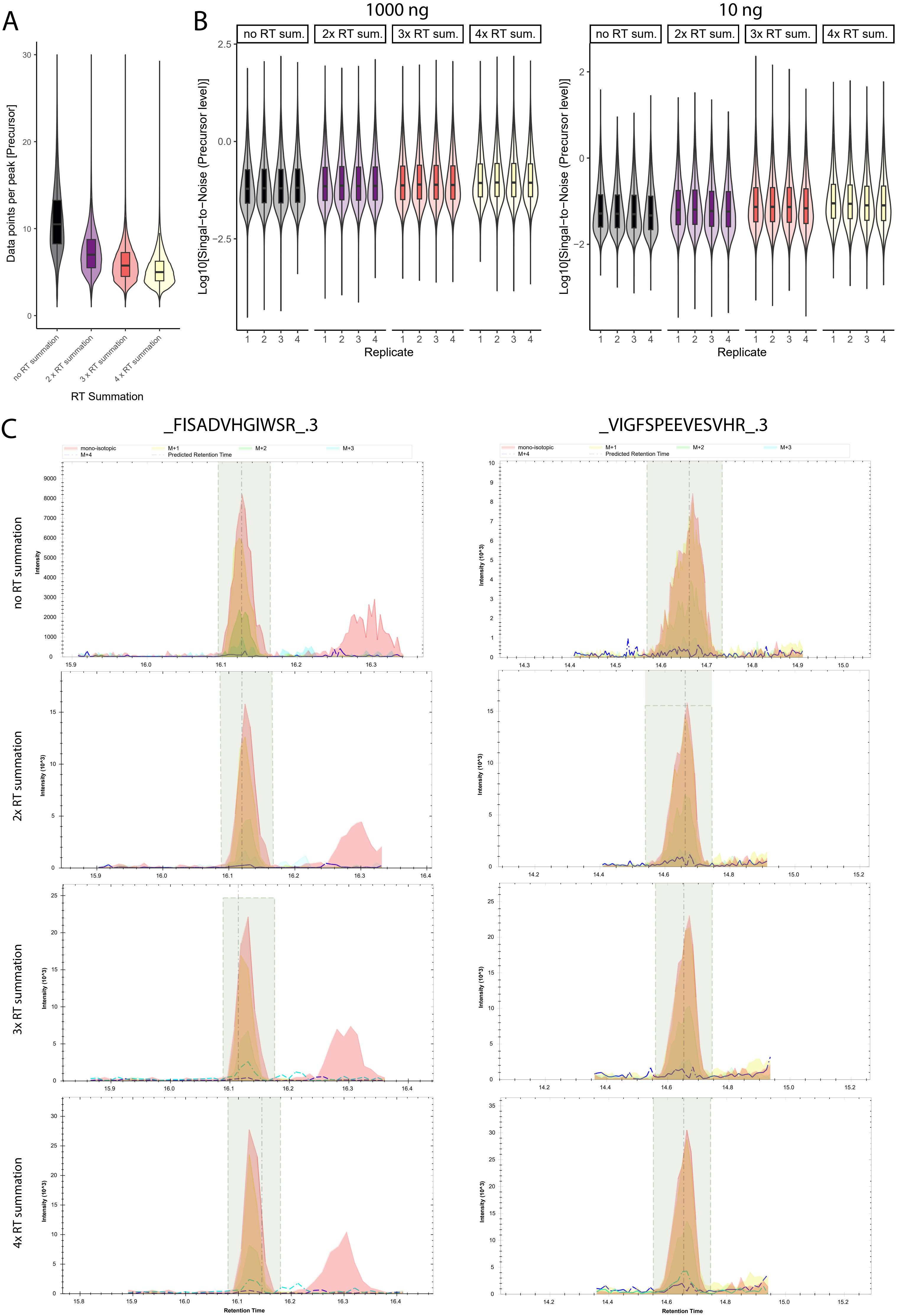
A
B
1000 ng
10 ng
no RT sum.
2x RT sum.
3x RT sum.
4x RT sum.
Data points per peak [Precursor]
RT Summation
no RT summation
2 x RT summation
3 x RT summation
4 x RT summation
Log10[Singal-to-Noise (Precursor level)]
Replicate
C
FISADVHGIWSR.3
VIGFSPEEVESVHR.3
mono-isotopic
M+1
M+2
M+3
M+4
Predicted Retention Time
Intensity
Intensity (10^3)
no RT summation
2x RT summation
3x RT summation
4x RT summation
Retention Time

Supplementary Figure 3. A: Data point per peak distribution on precursor level for a 2-slice diagonal-PASEF method post indicated retention time summation. Mean DPPP for each precursor across all replicates shown (n = 4)**. B:** Log10 signal-to-noise ratio for all detected precursors of the method from A across different retention time summation values for 1000 ng (left) or 10 ng (right) loading of a HeLa sample. Individual boxplots and violin plots depict different replicates. **C:** Extracted ion chromatogram (XIC) of two selected precursors identified for an acquisition of 1000 ng of HeLa using the same method as from A subjected to the indicated retention time summation. Only data from replicate number two shown which was representative of all acquired replicates. All Boxplots indicate the inter-quartile range (IQR) from the lower quartile to the upper quartile. Central line indicates the median value of the population and whiskers indicate the 1.5 x IQR. Median CV is indicated above the plot. Outliers are not shown to aid the visual interpretation of the data.

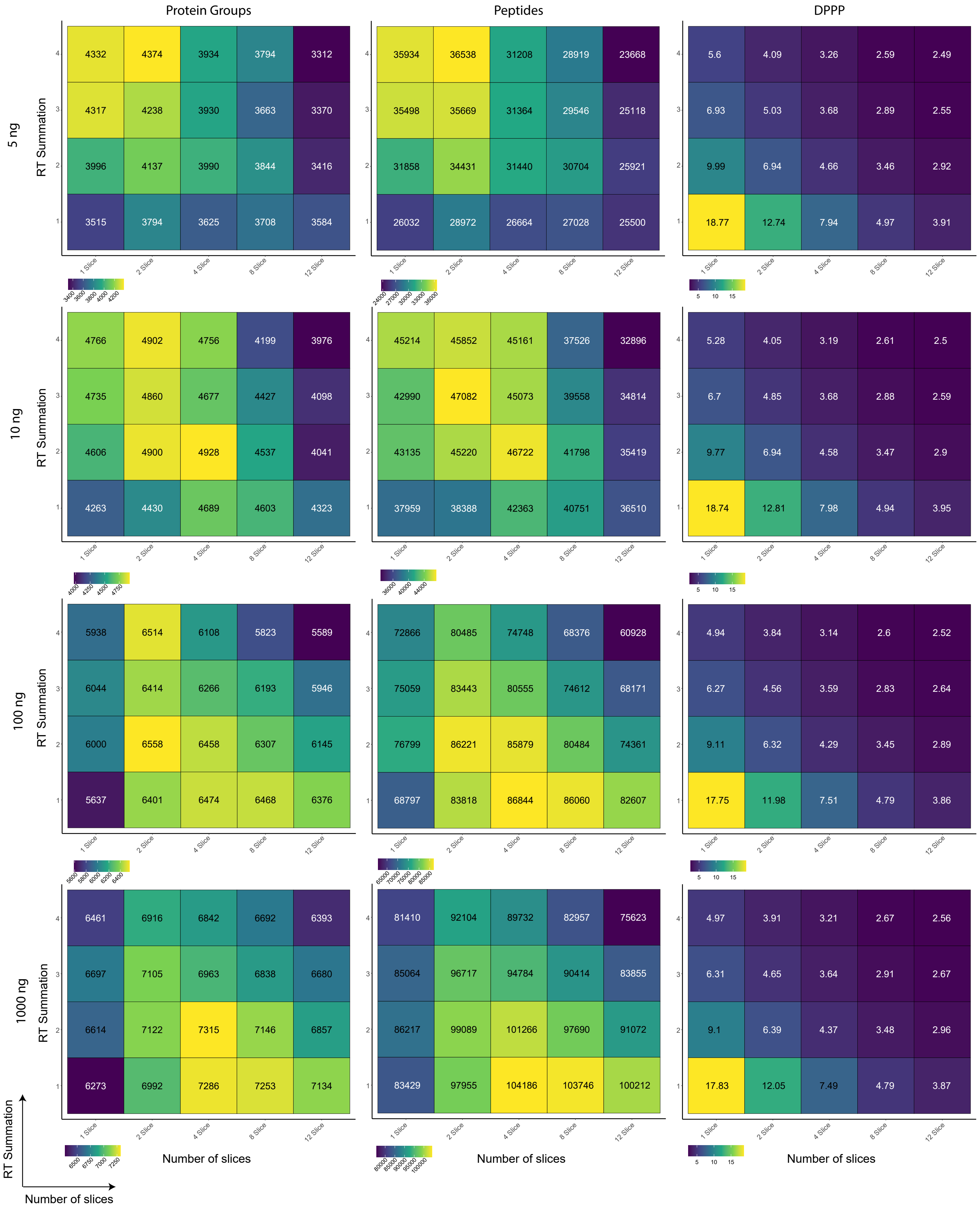
Protein Groups
Peptides
DPPP
5 ng
RT Summation
4332 4374 3934 3794 3312
4317 4238 3930 3663 3370
3996 4137 3990 3844 3416
3515 3794 3625 3708 3584
35934 36538 31208 28919 23668
35498 35669 31364 29546 25118
31858 34431 31440 30704 25921
26032 28972 26664 27028 25500
5.6 4.09 3.26 2.59 2.49
6.93 5.03 3.68 2.89 2.55
9.99 6.94 4.66 3.46 2.92
18.77 12.74 7.94 4.97 3.91
1 Slice 2 Slice 4 Slice 8 Slice 12 Slice
3400 3600 3800 4000 4200
24000 27000 30000 33000 36000
5 10 15
10 ng
RT Summation
4766 4902 4756 4199 3976
4735 4860 4677 4427 4098
4606 4900 4928 4537 4041
4263 4430 4689 4603 4323
45214 45852 45161 37526 32896
42990 47082 45073 39558 34814
43135 45220 46722 41798 35419
37959 38388 42363 40751 36510
5.28 4.05 3.19 2.61 2.5
6.7 4.85 3.68 2.88 2.59
9.77 6.94 4.58 3.47 2.9
18.74 12.81 7.98 4.94 3.95
4000 4250 4500 4750
36000 40000 44000
100 ng
RT Summation
5938 6514 6108 5823 5589
6044 6414 6266 6193 5946
6000 6558 6458 6307 6145
5637 6401 6474 6468 6376
72866 80485 74748 68376 60928
75059 83443 80555 74612 68171
76799 86221 85879 80484 74361
68797 83818 86844 86060 82607
4.94 3.84 3.14 2.6 2.52
6.27 4.56 3.59 2.83 2.64
9.11 6.32 4.29 3.45 2.89
17.75 11.98 7.51 4.79 3.86
5600 5800 6000 6200 6400
65000 70000 75000 80000 85000
1000 ng
RT Summation
6461 6916 6842 6692 6393
6697 7105 6963 6838 6680
6614 7122 7315 7146 6857
6273 6992 7286 7253 7134
81410 92104 89732 82957 75623
85064 96717 94784 90414 83855
86217 99089 101266 97690 91072
83429 97955 104186 103746 100212
4.97 3.91 3.21 2.67 2.56
6.31 4.65 3.64 2.91 2.67
9.1 6.39 4.37 3.48 2.96
17.83 12.05 7.49 4.79 3.87
6500 6750 7000 7250
80000 85000 90000 95000 100000
Number of slices
RT Summation
Number of slices

Supplementary Figure 4. Results from systematic testing of the retention time summation on all diagonal-PASEF methods. Average protein groups (left), peptides (center) and data points per peak (DPPP) (right) across four replicates are shown for each tested diagonal-PASEF method (x-axis) against the utilized retention time summation value (y-axis). Results are shown for the indicated loading from 5 (top) to 1000 ng (bottom). Average identifications are indicated within the tiles.

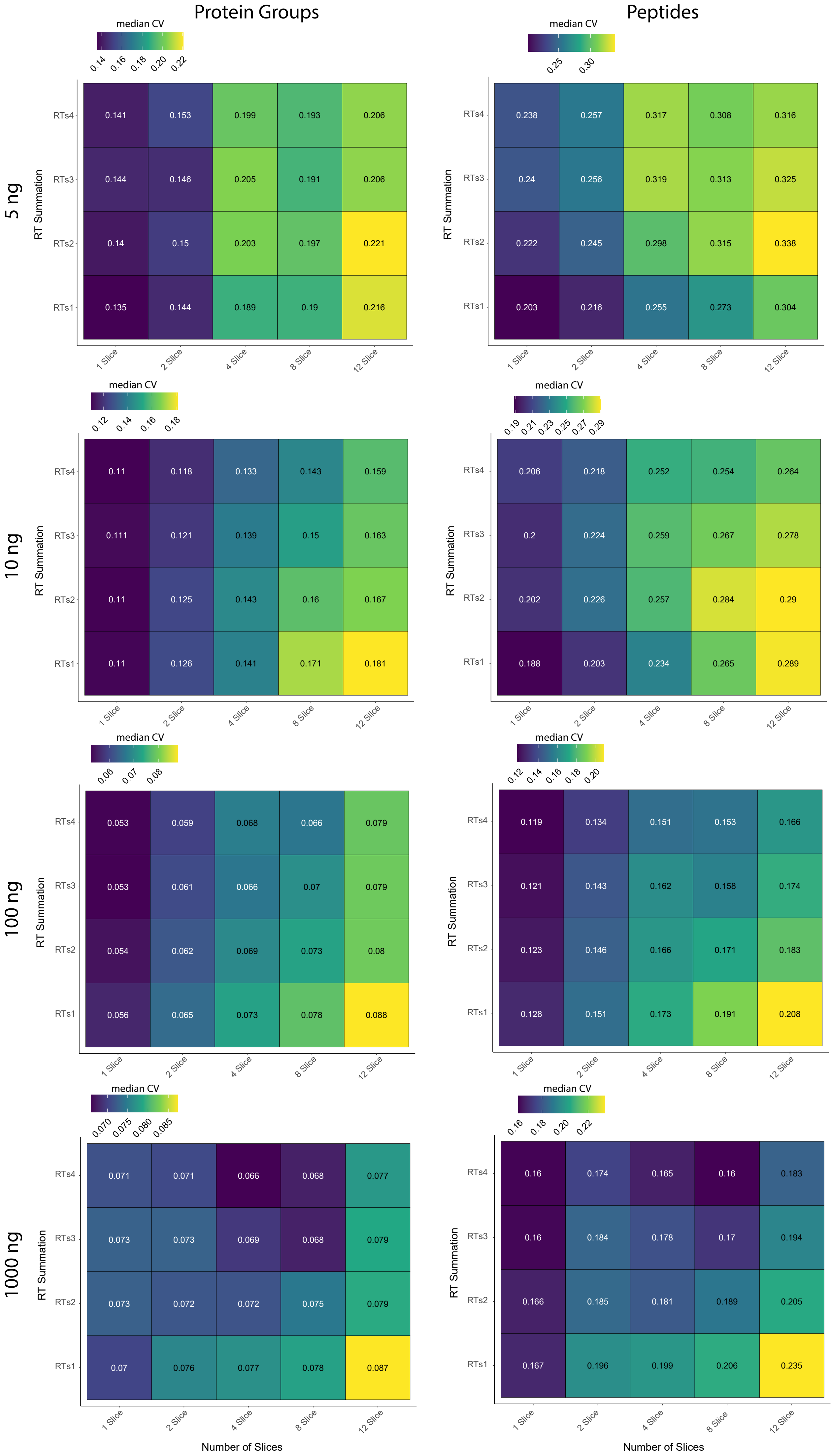
Protein Groups
Peptides
5 ng
10 ng
100 ng
1000 ng
RT Summation
median CV
RTs4
RTs3
RTs2
RTs1
1 Slice
2 Slice
4 Slice
8 Slice
12 Slice
Number of Slices
0.14 0.16 0.18 0.20 0.22
0.141 0.153 0.199 0.193 0.206
0.144 0.146 0.205 0.191 0.206
0.14 0.15 0.203 0.197 0.221
0.135 0.144 0.189 0.19 0.216
0.25 0.30
0.238 0.257 0.317 0.308 0.316
0.24 0.256 0.319 0.313 0.325
0.222 0.245 0.298 0.315 0.338
0.203 0.216 0.255 0.273 0.304
0.12 0.14 0.16 0.18
0.11 0.118 0.133 0.143 0.159
0.111 0.121 0.139 0.15 0.163
0.11 0.125 0.143 0.16 0.167
0.11 0.126 0.141 0.171 0.181
0.19 0.21 0.23 0.25 0.27 0.29
0.206 0.218 0.252 0.254 0.264
0.2 0.224 0.259 0.267 0.278
0.202 0.226 0.257 0.284 0.29
0.188 0.203 0.234 0.265 0.289
0.06 0.07 0.08
0.053 0.059 0.068 0.066 0.079
0.053 0.061 0.066 0.07 0.079
0.054 0.062 0.069 0.073 0.08
0.056 0.065 0.073 0.078 0.088
0.12 0.14 0.16 0.18 0.20
0.119 0.134 0.151 0.153 0.166
0.121 0.143 0.162 0.158 0.174
0.123 0.146 0.166 0.171 0.183
0.128 0.151 0.173 0.191 0.208
0.070 0.075 0.080 0.085
0.071 0.071 0.066 0.068 0.077
0.073 0.073 0.069 0.068 0.079
0.073 0.072 0.072 0.075 0.079
0.07 0.076 0.077 0.078 0.087
0.16 0.18 0.20 0.22
0.16 0.174 0.165 0.16 0.183
0.16 0.184 0.178 0.17 0.194
0.166 0.185 0.181 0.189 0.205
0.167 0.196 0.199 0.206 0.235

Supplementary Figure 5: Median CV values on protein group (left) or peptide (right) level from systematic testing of all investigated diagonal-PASEF methods (x-axis) against the utilized retention time summation value (y-axis). Results are shown for the indicated loading from 5 (top) to 1000 ng (bottom). Median CV was computed across all four replicates of each acquisition and applied RT summation on indicated analyte level and is depicted in center of the tile.

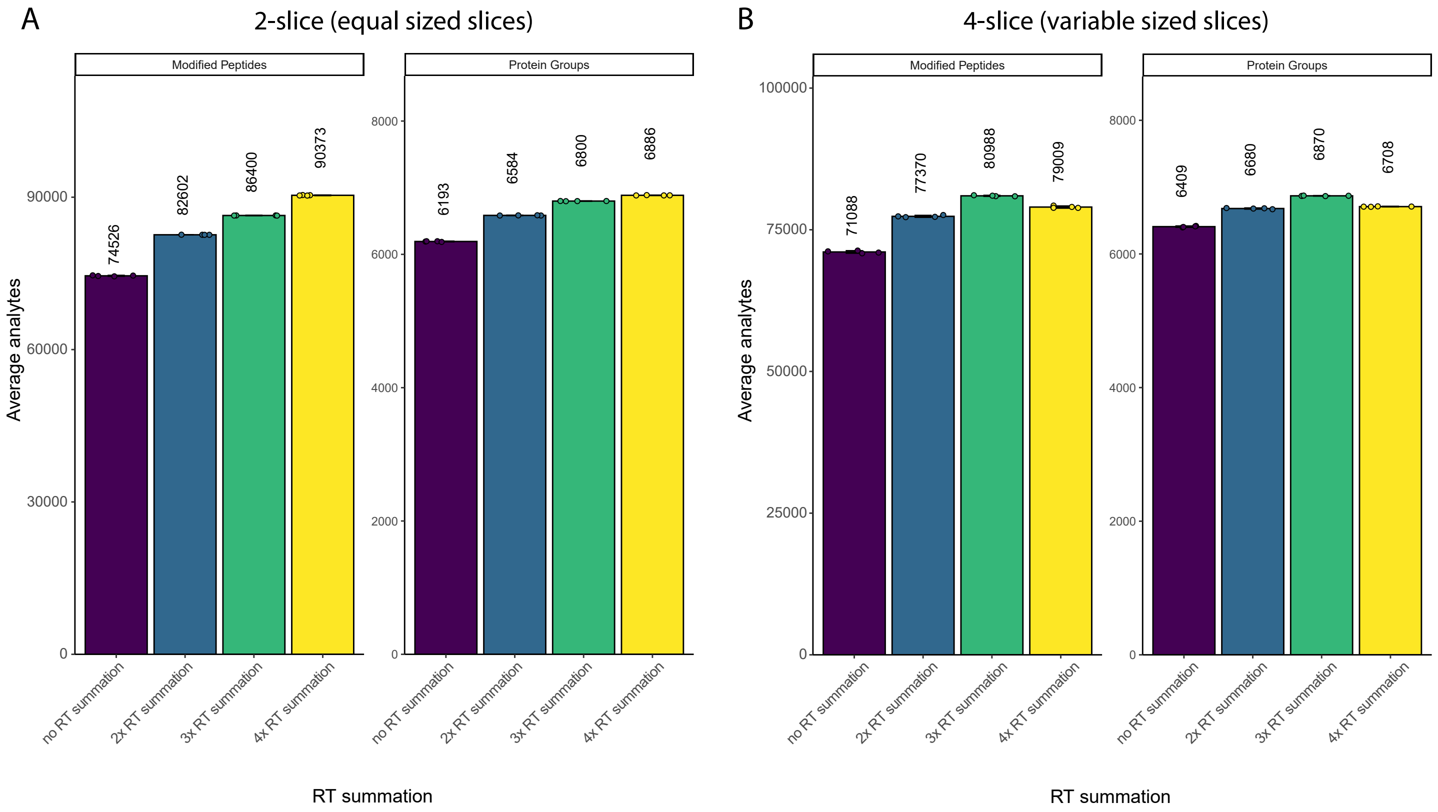
A
2-slice (equal sized slices)
Modified Peptides
Protein Groups
Average analytes
74526
82602
86400
90373
6193
6584
6800
6886
no RT summation
2x RT summation
3x RT summation
4x RT summation
RT summation
B
4-slice (variable sized slices)
71088
77370
80988
79009
6409
6680
6870
6708

Supplementary Figure 6: A-B: Re-analysis of previously published diagonal-PASEF data and application of RT summation. 2-slice diagonal-PASEF acquisitions with equal width (**A**) or 4-slice diagonal-PASEF acquisitions with variable width (**B**) from 30 SPD data from Skowronek et al Nature Protocols (2025) analyzed in Spectronaut 20 with indicated RT summation. Each datapoint indicates an individual replicate (n = 4). Mean and standard deviation of mean shown. Values above bars indicate average analytes.

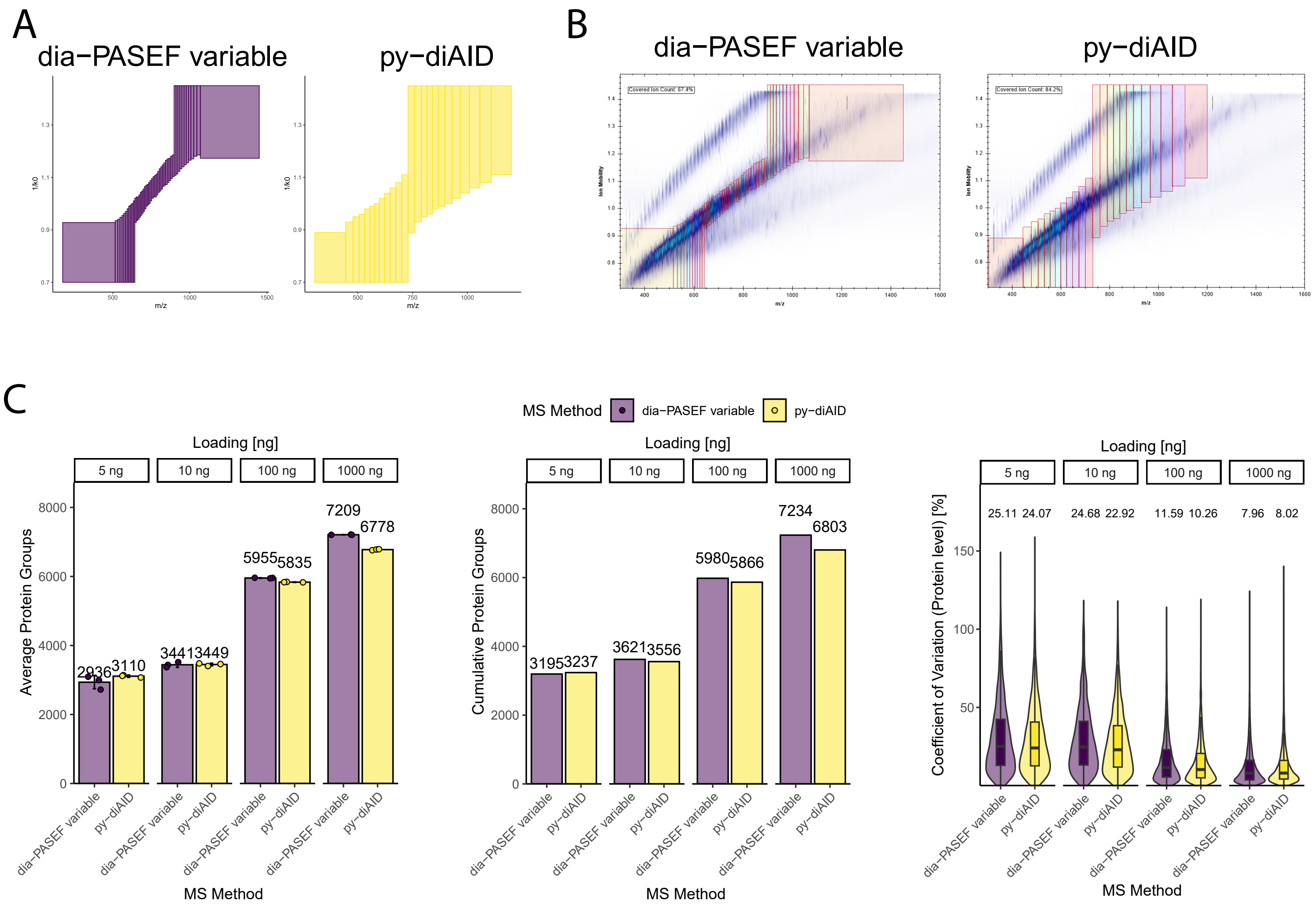

A
dia−PASEF variable
py−diAID
B
dia−PASEF variable
py−diAID
C
MS Method
dia−PASEF variable
py−diAID
Loading [ng]
5 ng
10 ng
100 ng
1000 ng
Average Protein Groups
2936
3110
3441
3449
5955
5835
7209
6778
Cumulative Protein Groups
3195
3237
3621
3556
5980
5866
7234
6803
Coefficient of Variation (Protein level) [%]
25.11
24.07
24.68
22.92
11.59
10.26
7.96
8.02
MS Method

Supplementary Figure 7. A-C: Comparison of ‘dia-PASEF variable’ method applied in this study against a method generated through the py-diAID tool. **A**: Visual representation of both methods. Each method consisted of 12 PASEF slices resolved over a different number of DIA windows and covered the same 1/k_0 range (0.7 – 1.45). **B:** Representative visualization of 'dia-PASEF variable (left) or py-diAID optimized dia-PASEF (right) acquisition scheme over all ions detected in a 17-minute gradient. Visualization was generated with Spectonaut using the ‘Ion mobility overview’ plot from replicate number two. **C:** Results from benchmarking of the dia-PASEF variable against the py-diAID method at the indicated loading amount. Left: Average protein group identifications from three replicates. Data points indicate individual acquisitions. Error bars represent standard deviation from the mean. Middle: Cumulative number of protein group identifications from a triplicate injections acquiring the indicated loading amount. Right: Coefficient of variation (CV) on protein group level for both tested methods at the indicated loading amount. Median CV for each method and loading is shown above the violin. All Boxplots indicate the inter-quartile range (IQR) from the lower quartile to the upper quartile. Central line indicates the median value of the population and whiskers indicate the 1.5 x IQR. Median CV is indicated above the plot. Outliers are not shown to aid the visual interpretation of the data.

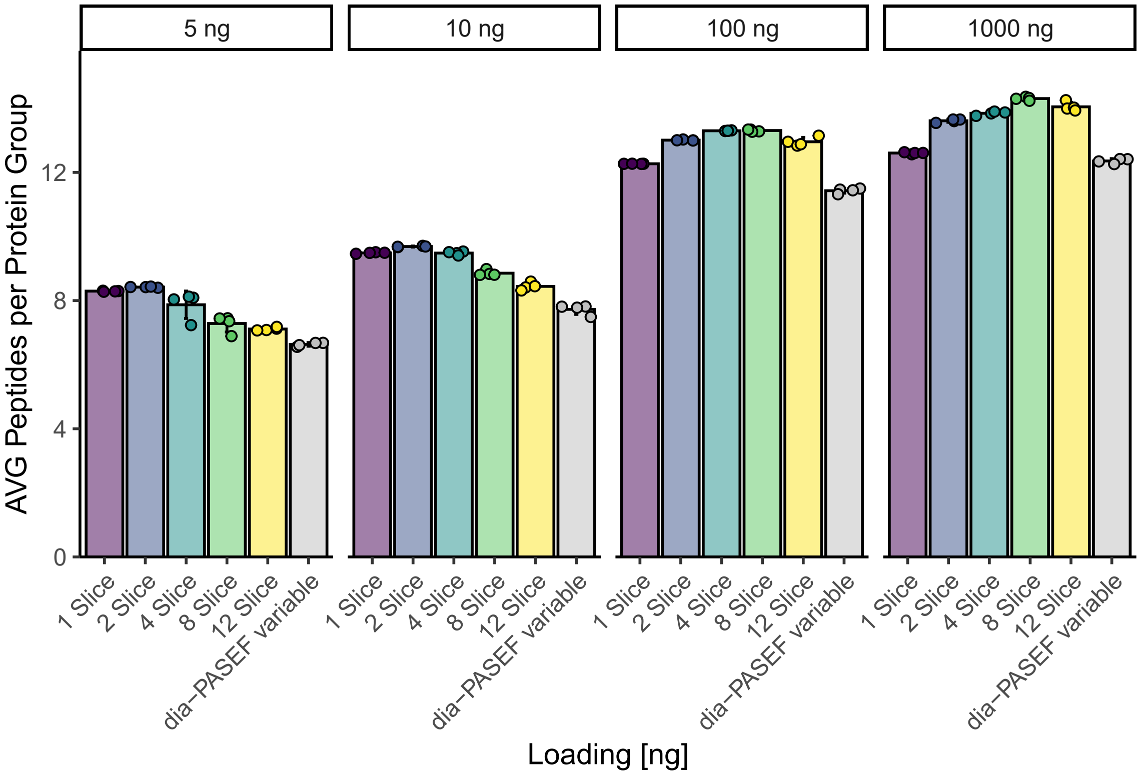

5 ng
10 ng
100 ng
1000 ng
AVG Peptides per Protein Group
0
4
8
12
1 Slice
2 Slice
4 Slice
8 Slice
12 Slice
dia−PASEF variable
Loading [ng]

Supplementary Figure 8. Average number of peptide identifications per protein group for all tested dia-PASEF and diagonal-PASEF methods for the indicated loadings. The height of the bar represents the average value across quadruplicates with standard deviation around mean shown. Data points indicate individual replicates (n = 4). Error bars represent standard deviation of mean. The optimal retention time summation was applied to all diagonal-PASEF acquisitions as follows: 1-slice: 4xRTs, 2-slice: 3xRTs, 4-slice: 2xRTs, 8-slice: 1xRTs, 12-slice: 1xRTs.

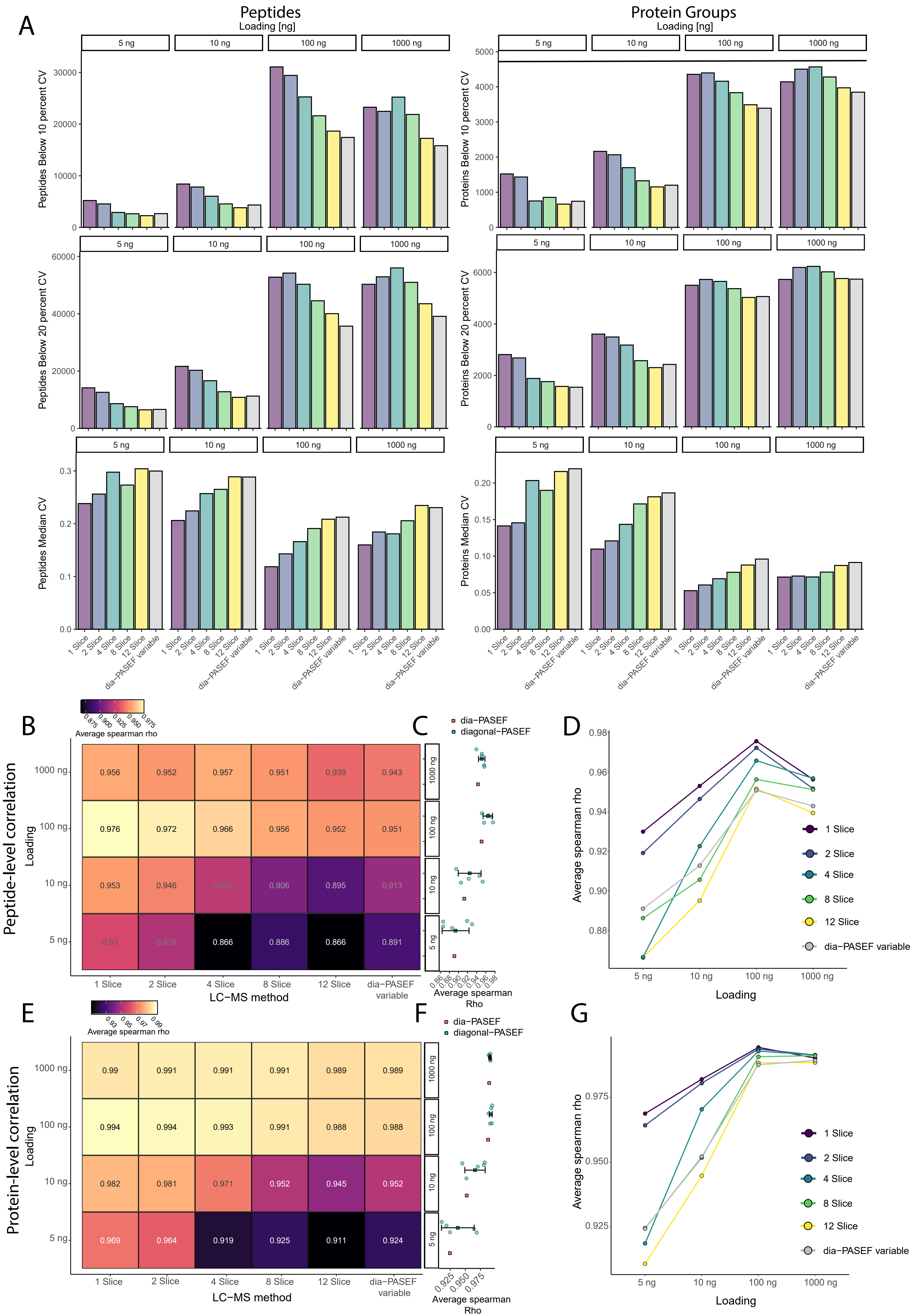
A
Peptides
Loading [ng]
Protein Groups
Loading [ng]
5 ng
10 ng
100 ng
1000 ng
Peptides Below 10 percent CV
Proteins Below 10 percent CV
Peptides Below 20 percent CV
Proteins Below 20 percent CV
Peptides Median CV
Proteins Median CV
1 Slice
2 Slice
4 Slice
8 Slice
12 Slice
dia−PASEF variable
B
Average spearman rho
Peptide-level correlation
Loading
1000 ng
0.956
0.952
0.957
0.951
0.939
0.943
100 ng
0.976
0.972
0.966
0.956
0.952
0.951
10 ng
0.953
0.946
0.923
0.906
0.895
0.913
5 ng
0.93
0.919
0.866
0.886
0.866
0.891
LC−MS method
C
dia−PASEF
diagonal−PASEF
Average spearman Rho
D
Average spearman rho
Loading
E
Average spearman rho
Protein-level correlation
Loading
1000 ng
0.99
0.991
0.991
0.991
0.989
0.989
100 ng
0.994
0.994
0.993
0.991
0.988
0.988
10 ng
0.982
0.981
0.971
0.952
0.945
0.952
5 ng
0.969
0.964
0.919
0.925
0.911
0.924
LC−MS method
F
dia−PASEF
diagonal−PASEF
Average spearman Rho
G
Average spearman rho
Loading

Supplementary Figure 9. A: Results from the loading ramp experiment for all tested diagonal- or dia-PASEF methods and loadings. Peptide (left) or Protein group (right) identifications below 10% CV (top) or 20% CV (center) or median CV (bottom) are shown. **B**: Average Spearman-based rank correlation rho-value on peptide level of all replicates tested against each other for each tested acquisition method (x-axis) and loading (y-axis). Each tile represents the average Spearman correlation rho value from all correlations for this method which is also indicated in the tile center. **C**: Average Spearman rho value from B stratified by acquisition-type. For diagonal-PASEF: Central datapoint indicates the mean correlation rho value for across all tested methods. Error bars indicate standard deviation around mean rho value. Individual datapoints indicate correlation values from individual methods (Average from all replicates shown). **D**: Average Spearman rho value from B visualized across different loadings for all tested methods. Individual acquisition methods are color coded. **E-G**: Same analyses as depicted in B-D but on protein group level. For all panels, the optimal retention time summation was applied to all diagonal-PASEF acquisitions as follows: 1-slice: 4xRTs, 2-slice: 3xRTs, 4-slice: 2xRTs, 8-slice: 1xRTs, 12-slice: 1xRTs.

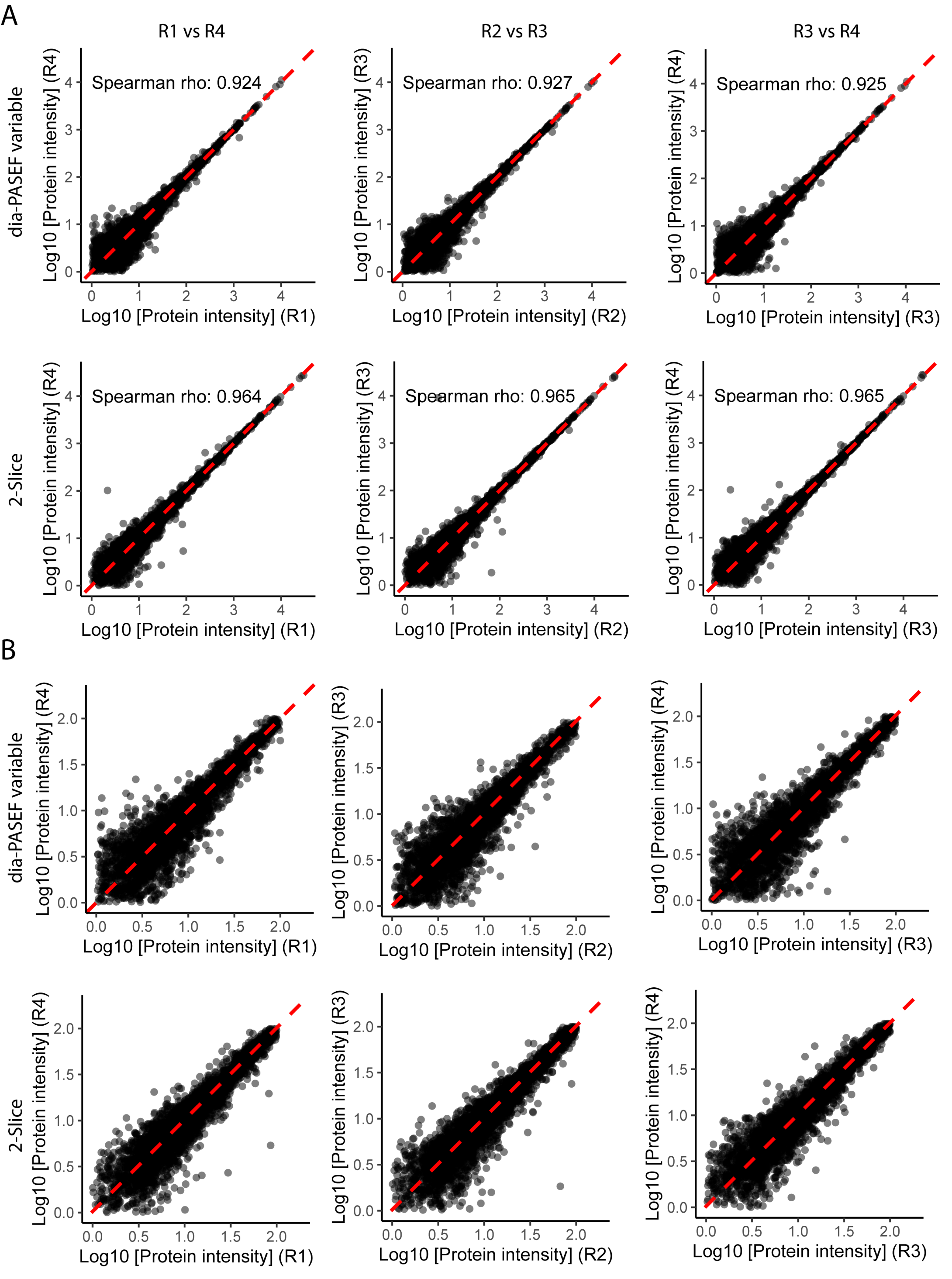
A
R1 vs R4
R2 vs R3
R3 vs R4
dia-PASEF variable
2-Slice
Spearman rho: 0.924
Spearman rho: 0.927
Spearman rho: 0.925
Spearman rho: 0.964
Spearman rho: 0.965
Spearman rho: 0.965
Log10 [Protein intensity] (R1)
Log10 [Protein intensity] (R2)
Log10 [Protein intensity] (R3)
Log10 [Protein intensity] (R4)
Log10 [Protein intensity] (R3)
Log10 [Protein intensity] (R4)
B
dia-PASEF variable
2-Slice
Log10 [Protein intensity] (R1)
Log10 [Protein intensity] (R2)
Log10 [Protein intensity] (R3)
Log10 [Protein intensity] (R4)
Log10 [Protein intensity] (R3)
Log10 [Protein intensity] (R4)

Supplementary Figure 10. A: Representative Spearman-based rank correlations of individual selected replicates for the dia-PASEF variable (top) or the 2-slice diagonal-PASEF (bottom) method on the protein level for 5 ng HeLa acquisitions. The 2-slice diagonal-PASEF method was subjected to a retention time summation of 3. Each data point represents a unique protein. Dashed red line represents the linear regression between the two indicated replicates. Correlation values from Spearman-based rank correlation. **B:** Same as in A but for proteins with log10(protein quantity) <= 2 to visualize low-abundant proteins. Selected replicate-to-replicate correlations are representative of correlations of all tested replicates.

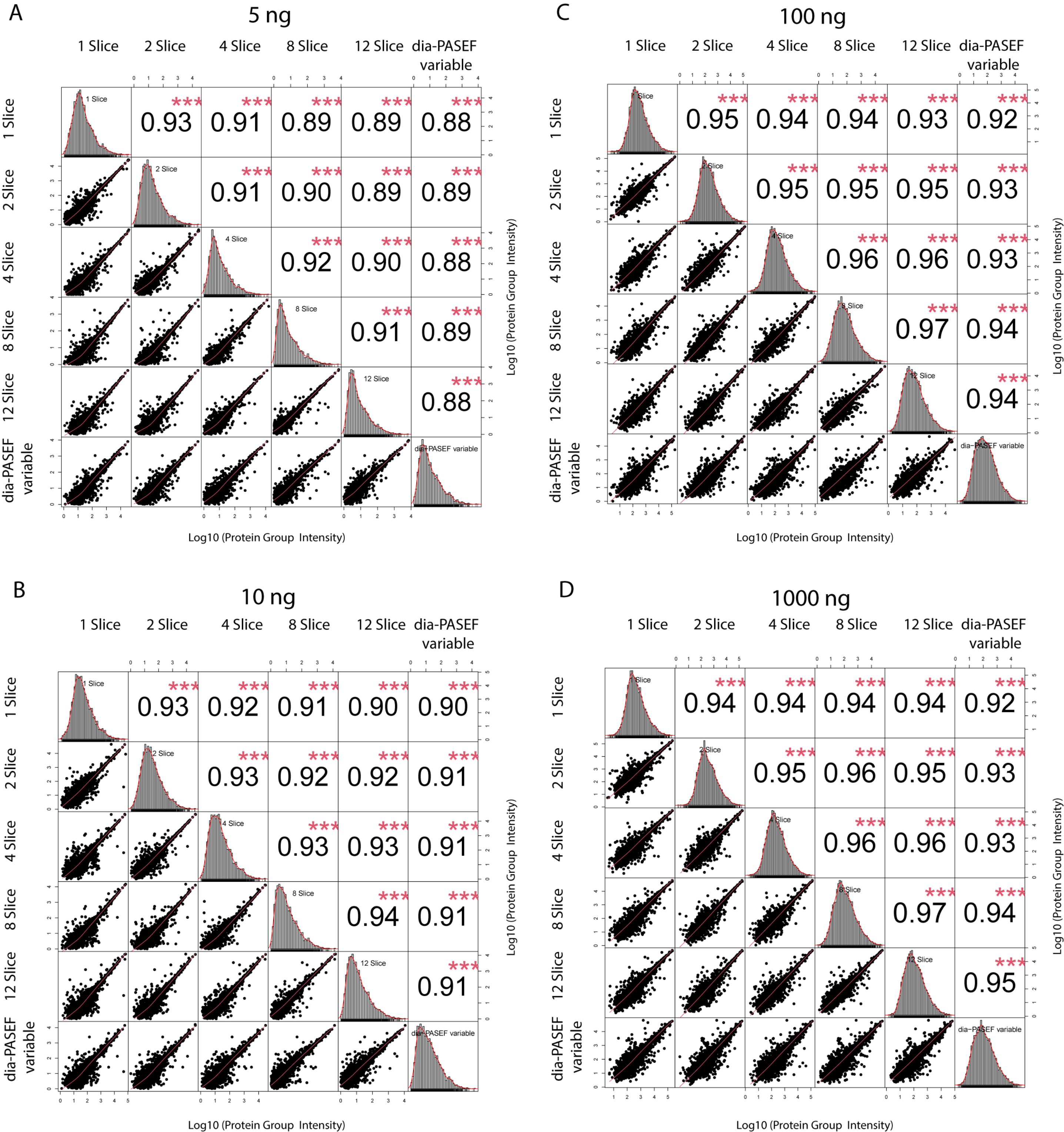

A
5 ng
1 Slice
2 Slice
4 Slice
8 Slice
12 Slice
dia-PASEF variable
0.93
0.91
0.89
0.89
0.88
0.91
0.90
0.89
0.89
0.92
0.90
0.88
0.91
0.89
0.88
Log10 (Protein Group Intensity)
B
10 ng
1 Slice
2 Slice
4 Slice
8 Slice
12 Slice
dia-PASEF variable
0.93
0.92
0.91
0.90
0.90
0.93
0.92
0.92
0.91
0.93
0.93
0.91
0.94
0.91
0.91
Log10 (Protein Group Intensity)
C
100 ng
1 Slice
2 Slice
4 Slice
8 Slice
12 Slice
dia-PASEF variable
0.95
0.94
0.94
0.93
0.92
0.95
0.95
0.95
0.93
0.96
0.96
0.93
0.97
0.94
0.94
Log10 (Protein Group Intensity)
D
1000 ng
1 Slice
2 Slice
4 Slice
8 Slice
12 Slice
dia-PASEF variable
0.94
0.94
0.94
0.94
0.92
0.95
0.96
0.95
0.93
0.96
0.96
0.93
0.97
0.94
0.95
Log10 (Protein Group Intensity)

Supplementary Figure 11. A-D: Spearman-based **c**orrelation analysis of all tested acquisition methods on protein level against each other at 5 ng (**A**), 10 ng (**B**), 100 ng (**C)** or 1000 ng (**D**) of loading. For each correlation analysis the mean protein quantity was computed across all four replicates of each method and loading. In top right panels the Spearman rho values and the correlation significance level are shown for the indicated correlation. Diagonal panels show the protein distribution for each acquisition method. Bottom right panels show the individual proteins that were correlated in each correlation analysis. Each data point represents a unique protein. Significance levels of correlations as follows: * $p < 0.05$, ** $p < 0.01$, *** $p < 0.001$. For all panels, the optimal retention time summation was applied to all diagonal-PASEF acquisitions as follows: 1-slice: 4xRTs, 2-slice: 3xRTs, 4-slice: 2xRTs, 8-slice: 1xRTs, 12-slice: 1xRTs.

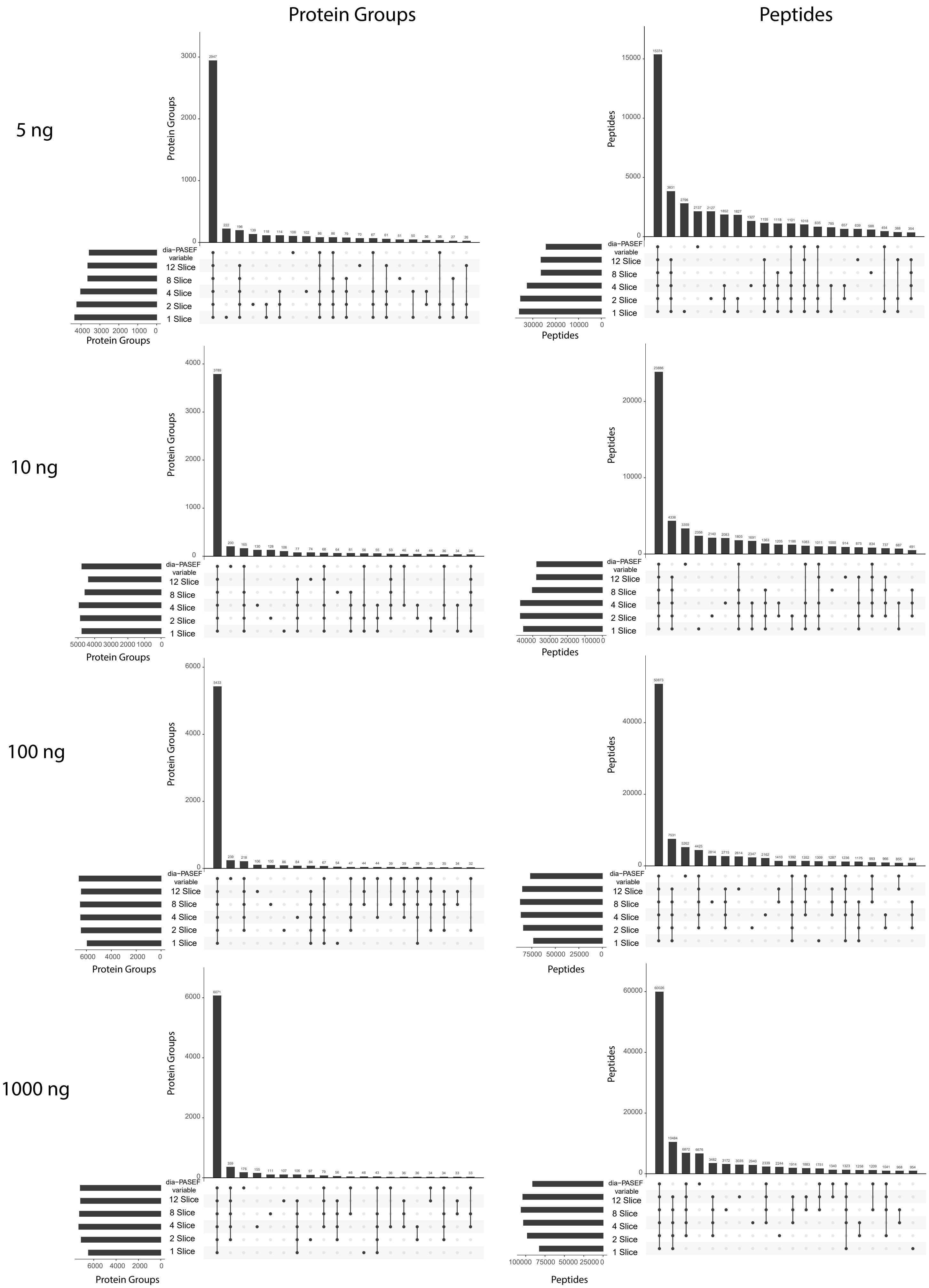
Protein Groups
Peptides
5 ng
10 ng
100 ng
1000 ng
dia−PASEF variable
12 Slice
8 Slice
4 Slice
2 Slice
1 Slice
Protein Groups
Peptides

Supplementary Figure 12. Upset plots on protein (left) or peptide (right) level for all tested acquisition methods at the indicated loading. Top panels show the number of analytes in each interaction group from bottom panels. Left panels show the cumulative number of analytes from each acquisition method. For all panels, the optimal retention time summation was applied to all diagonal-PASEF acquisitions as follows: 1-slice: 4xRTs, 2-slice: 3xRTs, 4-slice: 2xRTs, 8-slice: 1xRTs, 12-slice: 1xRTs.

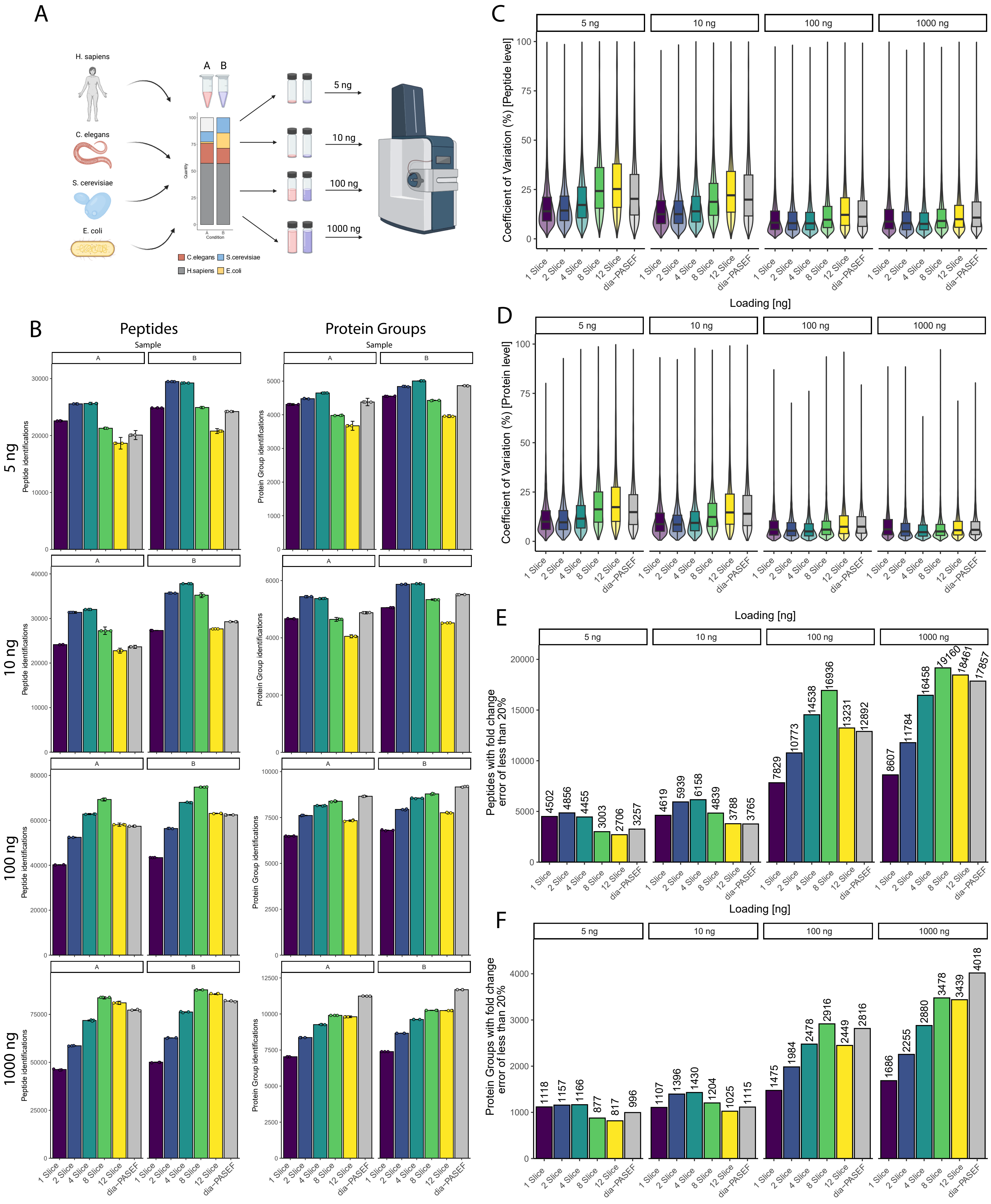

A
H. sapiens
C. elegans
S. cerevisiae
E. coli
A B
5 ng
10 ng
100 ng
1000 ng
Quantity
Condition
C.elegans S.cerevisiae
H.sapiens E.coli
B
Peptides
Protein Groups
Sample
5 ng
10 ng
100 ng
1000 ng
Peptide identifications
Protein Group identifications
1 Slice
2 Slice
4 Slice
8 Slice
12 Slice
dia–PASEF
C
Coefficient of Variation (%) [Peptide level]
Loading [ng]
D
Coefficient of Variation (%) [Protein level]
Loading [ng]
E
Peptides with fold change error of less than 20%
5 ng: 4502 4856 4455 3003 2706 3257
10 ng: 4619 5939 6158 4839 3788 3765
100 ng: 7829 10773 14538 16936 13231 12892
1000 ng: 8607 11784 16458 19160 18461 17857
Loading [ng]
F
Protein Groups with fold change error of less than 20%
5 ng: 1118 1157 1166 877 817 996
10 ng: 1107 1396 1430 1204 1025 1115
100 ng: 1475 1984 2478 2916 2449 2816
1000 ng: 1686 2255 2880 3478 3439 4018

Supplementary Figure 13: A: Experimental approach conducted for the controlled quantitative experiment (CQE) shown in this study and composition of samples “A” and “B” based on protein amount. **B:** Average peptide (left) or protein group (right) identifications across tested acquisition methods and loadings. Mean and standard deviation of mean shown. Each datapoint indicates identification from a single replicate (n = 3). **C-D**: Coefficient of variation (CV) on peptide (C) or protein (D) level across all replicates across tested acquisition methods and loadings. CV values were averaged between samples A and B. Only a CV range between 0 and 100% is shown to aid the visual interpretation of the data. **E-F**: **:** Number of peptides (**E**) or protein groups (**F**) quantified with a fold-change error of less than 20%. Values indicate the height of the bar. For all panels, the optimal retention time summation was applied to all diagonal-PASEF acquisitions as follows: 1-slice: 4xRTs, 2-slice: 3xRTs, 4-slice: 2xRTs, 8-slice: 1xRTs, 12-slice: 1xRTs.

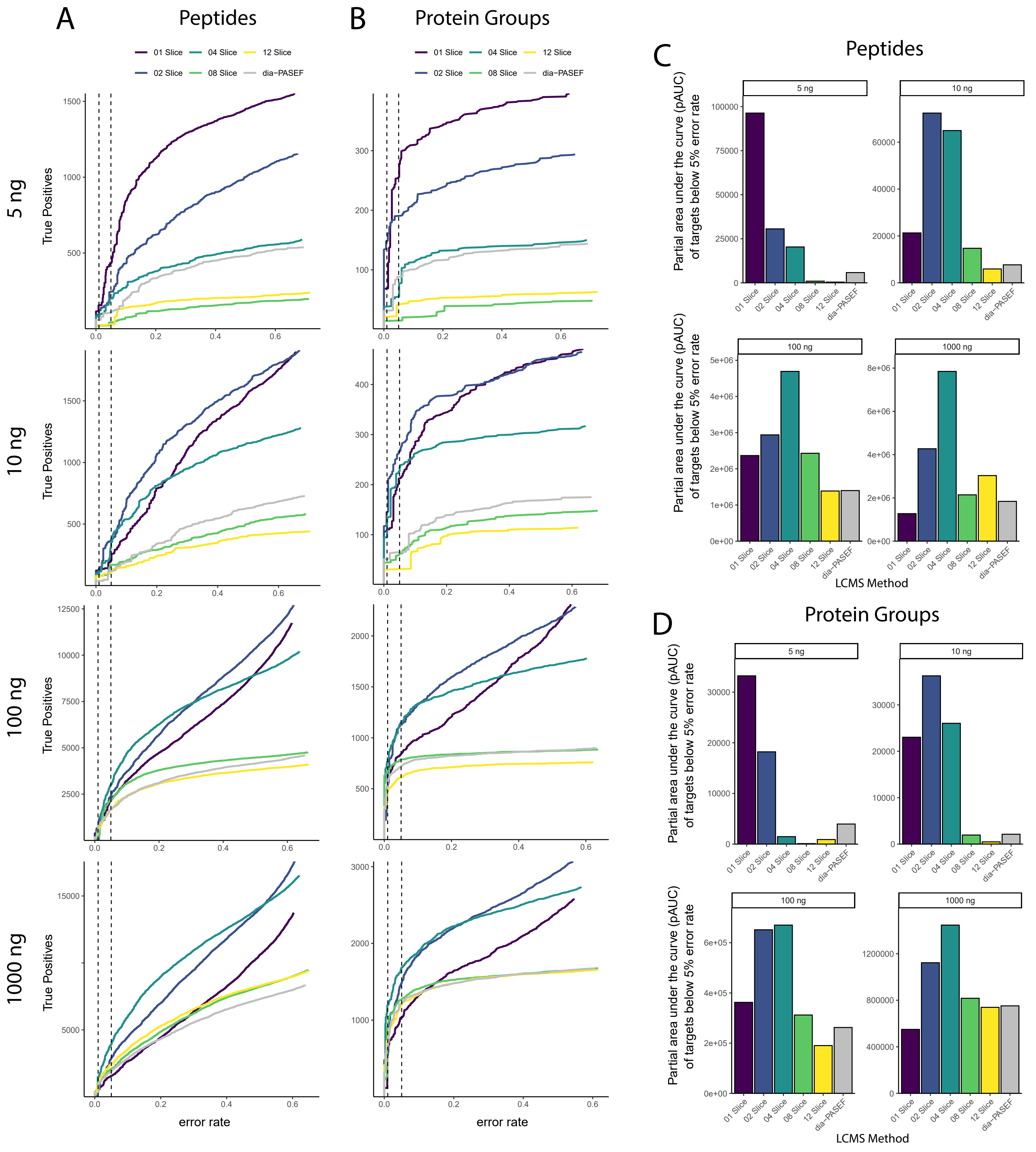
A
Peptides
B
Protein Groups
01 Slice
02 Slice
04 Slice
08 Slice
12 Slice
dia−PASEF
5 ng
10 ng
100 ng
1000 ng
True Positives
error rate
C
Peptides
Partial area under the curve (pAUC) of targets below 5% error rate
LCMS Method
D
Protein Groups
Partial area under the curve (pAUC) of targets below 5% error rate
LCMS Method

Supplementary Figure 14: A, B: True positive (TP) identifications over the error rate of candidates shown for diagonal-PASEF and dia-PASEF methods on peptide (**A**) or protein group (**B**) level across all tested loadings. Dashed vertical line indicate an error rate of 0.01 or 0.05.**C:** Partial area under the curve of true positive vs candidate length trajectory up to 5% error rate cut-off for selected diagonal-PASEF and dia-PASEF methods at all tested loadings on peptide (**C**) or protein group level (**D**).
